# Temporal Processing in the Visual Cortex of the Awake and Anesthetized Rat

**DOI:** 10.1523/ENEURO.0059-17.2017

**Published:** 2017-08-07

**Authors:** Ida E. J. Aasebø, Mikkel E. Lepperød, Maria Stavrinou, Sandra Nøkkevangen, Gaute Einevoll, Torkel Hafting, Marianne Fyhn

**Affiliations:** 1 Department of Biosciences, University of Oslo, Norway; 2 Institute of Basic Medical Sciences, University of Oslo, Norway; 3 Faculty of Science and Technology, Norwegian University of Life Sciences, Norway; 4 Department of Psychology, University of Oslo, Norway; 5 Department of Physics, University of Oslo, Norway

**Keywords:** anesthesia, awake, computational modeling, single units, temporal sequences, visual cortex

## Abstract

The activity pattern and temporal dynamics within and between neuron ensembles are essential features of information processing and believed to be profoundly affected by anesthesia. Much of our general understanding of sensory information processing, including computational models aimed at mathematically simulating sensory information processing, rely on parameters derived from recordings conducted on animals under anesthesia. Due to the high variety of neuronal subtypes in the brain, population-based estimates of the impact of anesthesia may conceal unit- or ensemble-specific effects of the transition between states. Using chronically implanted tetrodes into primary visual cortex (V1) of rats, we conducted extracellular recordings of single units and followed the same cell ensembles in the awake and anesthetized states. We found that the transition from wakefulness to anesthesia involves unpredictable changes in temporal response characteristics. The latency of single-unit responses to visual stimulation was delayed in anesthesia, with large individual variations between units. Pair-wise correlations between units increased under anesthesia, indicating more synchronized activity. Further, the units within an ensemble show reproducible temporal activity patterns in response to visual stimuli that is changed between states, suggesting state-dependent sequences of activity. The current dataset, with recordings from the same neural ensembles across states, is well suited for validating and testing computational network models. This can lead to testable predictions, bring a deeper understanding of the experimental findings and improve models of neural information processing. Here, we exemplify such a workflow using a Brunel network model.

## Significance Statement

As investigations of neural information processing have moved from anesthetized to alert animals, a question emerges on how we can compare data and interpret results from recordings from anesthetized animals. Previous efforts to investigate the impact of anesthesia on visual processing have frequently compared population responses from separate animals or experiments. The large diversity of neurons in the cortex demands comparisons within units and ensembles. Here, we followed units across states and found unpredictable and profound differences in the temporal dynamics of single units and ensembles between the anesthetized and awake states. To exemplify the importance of such a comparison, we demonstrate how such data can be used as a basis to test and develop models on network activity.

## Introduction

Despite the profound effect of anesthesia on the brain, neurons of the primary sensory cortices continue to respond to adequate stimuli under anesthesia. This has made anesthetized preparations attractive for studying sensory processing and has given insight into the fundamental principles of information processing. Wurtz et al. (1969) reported that neurons in the awake primate visual cortex respond similarly to visual stimuli to those of anesthetized cats and monkeys (Hubel and Wiesel, 1962, 1968). However, during natural vision, the relationship between visual stimulus and activity in visual cortex is less clear, indicating that cortical processing during behavior is heavily influenced by top-down processing and even input from other sensory modalities (Iurilli et al., 2012; Zhang et al., 2014). In the visual cortex of rodents, cortical activity is modulated by locomotion as well as behavioral state reflecting fundamental differences in cortical processing between the awake and anesthetized animal (Niell and Stryker, 2008, 2010; Keller et al., 2012; Ayaz et al., 2013). It is well known that anesthesia has profound effects on the brain, e.g., by suppression of neuron and glial cell activity (Greenberg et al., 2008; Schummers et al., 2008; Thrane et al., 2012; Vizuete et al., 2012) and effects on large-scale neuronal networks (Hentschke et al., 2005; Cimenser et al., 2011; Lewis et al., 2012; Pisauro et al., 2013; Bettinardi et al., 2015; Durand et al., 2016). Most of this previous work has been interexperimental comparisons with few experiments following the neurons between states. Consequently, less is known about how the activity of individual neurons in a cell ensemble is affected by the state transition between wakefulness and anesthesia. Considering the large diversity of neuronal subtypes in the cortex, investigations comparing separate populations of neurons recorded in each state can conceal how individual units in a population respond to the transition, since different neurons are sampled in the two populations.

Population activity may be unstructured (Kerr et al., 2005) or can be composed of default activity patterns (Luczak and MacLean, 2012). Neurons in auditory and somatosensory cortices fire in a sequential order during both spontaneous and sensory evoked activity (Luczak et al., 2007, 2009) perhaps reflecting common constraints from the cortical architecture (Luczak and MacLean, 2012). It remains unclear if such patterns are affected by anesthesia, since no previous investigations have followed the same population with single-unit recordings between states in the visual cortex. Moreover, the effect of anesthesia on stimulus-evoked latencies (Pisauro et al., 2013; Durand et al., 2016) may impact the temporal precision of responses and potentially the sequential patterns of activation. Temporal precision in neuronal firing is a characteristic of cortical activity in awake and behaving animals (Lee et al., 2005; Siapas et al., 2005) and of activity in the visual system (Mainen and Sejnowski, 1995; Reinagel and Reid, 2002), but how the temporal dynamics of ensembles of neurons in the visual cortex is affected by the transition from wakefulness to anesthesia, remains unresolved.

Using chronically implanted tetrodes in the rat visual cortex we conducted extracellular recordings of single units and local field potentials (LFPs) in response to visual stimulation and followed the same units during behavior, anesthesia, and after recovery from anesthesia. This method separates single units and can, with high temporal precision, follow their activity under different experimental conditions. In contrast to previous work comparing the transition between the awake and anesthetized states (Bayer, 2008; Greenberg et al., 2008) using calcium imaging of L2/3 visual cortex neurons, our approach have higher temporal dimension, relate changes to visual stimulation and cover unit sampling across all cortical depths. Furthermore, since the effects of anesthesia can be agent and area specific (Vahle-Hinz and Detsch, 2002), we compare three commonly used anesthetic agents to uncover the effects of anesthesia on unit activity in the visual cortex.

We quantified properties of the temporal activity of single units in local populations between wakefulness and anesthesia. Taken together, this compilation of results show that anesthesia alters temporal dynamics of neural ensembles in the visual cortex. Using a computational approach we further explored the hypothesis that increased inhibition in the network underlies the observed changes from awake to anesthetized states. With the standard and well-established Brunel-type network model consisting of leaky integrate-and-fire neurons (LIF; Brunel, 2000) we find that several of the salient experimental observations regarding differences between the awake and anesthetized states are qualitatively reproduced in the model.

## Materials and Methods

### Animals

Thirteen adult male Long Evans rats were bilaterally implanted with bundles of 16 wire electrodes in tetrode configuration (four tetrodes per hemisphere; Axona) for chronic recordings of neuronal activity in the primary visual cortex. Activity was recorded from the same units before, during, and after anesthesia while presenting visual stimuli. The same units were followed over the course of behavioral states for direct comparisons between states. After surgery, the animals were housed individually in transparent Plexiglas cages (35 × 40 × 40 cm) and provided with food and water *ad libitum*. They were kept on a 12/12 h light/dark cycle and testing occurred during the dark phase. All animal procedures were performed in accordance with guidelines from the Norwegian Animal Welfare Act and the European Convention for the Protection of Vertebrate Animals used for Experimental and Other Scientific Purposes.

### Electrode implantation

The rats were anesthetized with Isoflurane (1-3%, adjusted to obtain the appropriate surgical depth of anesthesia). Depth of anesthesia was assessed by monitoring heart rate and O_2_ saturation (%) in the blood (MouseSTAT, Kent Scientific). Body temperature was monitored and maintained at 37°C using homeothermic control system (Kent Scientific). The absence of withdrawal reflex after toe pinch was routinely checked. Analgesia during the procedure was secured by use of presurgically subcutaneous injections of temgesic (5 mg/kg) and Marcain adrenaline (0.14 ml/kg). Eyes were kept moist and protected by covering them in a layer of silicone oil. After Isoflurane induction the rats were fixed in a stereotaxic frame and the skull was exposed. Two bilateral craniotomies (1 mm in diameter) were drilled after vertical and horizontal alignment (according to bregma and lambda) of the head. The transverse sinus was visualized for accurate anterior-posterior coordinates. After a small incisions in the dura mater, microdrives with four tetrodes were shallowly implanted, one in each hemisphere, into the primary visual cortex, ∼300 µm below the dura mater at an angle of 30-40 degrees in the lateral to medial direction. Coordinates of implantations: 2.5-3.0 mm anterior to the midpoint of the transverse sinus and 4.8-5.2 lateral to the midline. The electrodes were secured to a microdrive (Axona). Seven small jeweler screws were attached to the skull and several applications of dental cement were used to fix the microdrives into place. Two of the screws were attached to reference electrodes on the microdrives, grounding each of the drives to the skull. To alleviate pain and minimize infections postsurgically, the rats were administered with rimadyl (5 mg/kg), penicillin (13.2 mg/kg), and convenia (8 mg/kg) on the three days following surgery.

### Behavior

During the awake recordings, the rats were allowed to move freely in a square glass enclosure (28 × 28 × 35 cm), with LCD monitors presenting the visual stimulus covering all four side walls. The monitors covered the rat visual field. The surrounding room was dark, thus the only visible stimulus to the rats was the stimulus presented on the screens. Numerous subsequent recordings with varied activity level were kept, such that each cell had recordings with maximum activity and minimum activity level. The rat’s activity level was monitored by tracking (rate, 50 Hz) the position of an infrared-light diode attached to the implant and correlated to neural activity recorded simultaneously. During the experimental sessions the animals were monitored closely and the session was discarded if the rat performed any grooming behavior or displayed signs of drowsing/sleeping.

To compare the neuron’s activity patterns in the awake and anesthetic states, awake recordings were immediately followed by recordings during anesthesia. The animals were anesthetized using three different regimes: Isoflurane only (1.5% which corresponds to the minimum alveolar concentration for adult rats; Mazze et al., 1985), Isoflurane with premedication of Dormicum (Isoflurane, 1%; Midazolam “Dormicum.” 1 mg/kg) or injections of a mixture of Ketamine and Xylazine (100 mg/kg Ketamine and 5 mg/kg Xylazine). The Isoflurane and Isoflurane/Dormicum conditions were initially tested with pilot studies to determine the lightest possible level of anesthesia. The Ketamine/Xylazine dose was chosen to match that used in Greenberg et al. (2008). Heart rate, blood oxygen saturation, and the LFP were continuously monitored. An effort was made to keep the animal as lightly anesthestized as possible in the Isoflurane conditions, adjusting the Isoflurane concentration according to the changes in certain physiologic parameters, such as reduced breathing rate, lower heartrate and presence of very low delta frequencies in the LFP. To avoid waking the rat from the light anesthesia, the loss of righting reflex was used to define the rat as unconscious instead of the toe pinch withdrawal reflex. Electrophysiological recordings were initiated 5 min after Isoflurane induction and 10 min after the injectable anesthetics. Before anethestic induction, droplets of tropicamide were applied to the eyes to widen the pupils. The eyelids were opened by applying a wax strip to the fur below the eyes and pulling the skin down. The head of the rat was placed 21 cm away from the screen on a raised platform, ensuring that the majority of the rats visual field was covered by the screen. Neural activity was recorded shortly after the anesthesia wore off (10–30 min after, recovery 1) and 24 h after anesthesia induction (recovery 2).

### Electrophysiology

Tetrodes for microdrives were prepared as described in Csicsvari et al. (1999) using 17-µm HM-L coated iridium/platinum electrode wire (California Fine Wire) twisted to form bundles of four electrodes. The tips of the electrodes were electroplated with platinum to reduce impedances to 100–200 kΩ at 1 kHz. Electrode arrays in the form of tetrodes can reliably be used to isolate several single units per tetrode by comparing the millisecond precise trace of the waveforms occurring on each electrode (Henze et al., 2000). The recorded signal from each electrode was amplified (5000–18,000×) band-pass filtered (0.8–6.7 kHz). Spikes were stored at 48 kHz (50 samples per wave form, eight bits per sample) using a 32-bit time stamp (96 Hz clock rate). The LFP was recorded single ended from one of the electrodes, low-pass filtered (500 Hz) and amplified 1000–2000×.

The recorded units from the electrodes were included without regard for their visual responsiveness, thus sampling nonselectively. All units that could be well isolated and displayed a consistent wave form and position signature throughout the recording period were included in the analyses. The depth-adjustable microdrive was lowered at increments of 50 µm to provide sampling cells from deeper cell layers. The tracks of the tetrodes were visualized histologically for anatomic position.

### Histology

At the end of experiments, the rats were anesthetized with pentobarbital (50 mg/kg) and perfused intracardially with 0.9% saline and 4% formaldehyde. The brains were stored in formaldehyde and placed in a 30% sucrose solution for 72 h before sectioning by a cryostat. Coronal sections (40 µm) were cut, mounted on glass slides, and stained for Nissl bodies with cresyl violet (Sigma-Aldrich). The tetrode tracks were measured and imaged with a light microscope (Axioplan microscope, Axiocam HRZ camera, AxioVision software and MosaiX, Zeiss). All electrode traces were verified to be localized within the visual cortex, based on cytoarchitectonic criteria. The recording location was extrapolated from deepest trace identified by histologic inspection of the sections and the tetrode-turning log. Shrinkage of the tissue was adjusted for.

### Spike sorting

Offline spike sorting was performed using graphical cluster-cutting software (Tint, Axona). Triggered spikes were assigned to clusters via the method of cluster cutting (Wilson and McNaughton, 1993; Skaggs et al., 1996), where a cell cluster is isolated from noise and other clusters on the basis of spike wave-shape and amplitude. Several evaluations of cluster quality were performed. First, a requirement of each cell cluster was that interspike interval histograms revealed few (<0.1%) or no spikes occurring within 2 ms of one another (Bruno and Simons, 2002). Second, the clusters need to be visibly isolated from the others and the separation distance between the clusters was quantified by calculating Mahalonobis distance (Harris et al., 2000). Third, the presence of a “common refractoriness,” i.e., an absence of spikes in the refractory period of two overlapping units, was used as an indication that two clusters belong to the same unit (Fee et al., 1996). Fourth, all cluster identification needed to clearly persist between the awake, anesthesia, and recovery states. Any ambiguity resulted in unit exclusion.

### Visual stimuli

The visual stimuli were presented on four monitors (Dell, Ultrasharp, 29 × 36 cm, 60-Hz refresh rate, mean luminance 70 cd/m^2^) positioned in a square closely surrounding the glass enclosure in the awake condition. The monitors were between 7–32 cm away from the rats head as he moved within the box, and distended 29 cm in height above him on all sides thus covering the rats visual space. In anesthesia the rat was placed 21 cm away from three of the screens, covering the visual field. The Psychophysics Toolbox extension in Matlab (Brainard, 1997; Pelli, 1997) was used to provide the visual stimuli, which consisted of drifting sinusoidal gratings where each orientation was presented for 0.5 s with a 0.5-s blank gray screen between each orientation. Eight orientations were used and the stimuli were repeated twelve times for each session with the orientation sequence randomly distributed. The Psychophysics toolbox program in Matlab was modified to report millisecond precise timestamps to the recording software on a separate computer. This ensured that initiation and termination of each stimulus was detected by the recording system and produced highly temporally accurate spike/stimulus data.

The same stimuli parameters were used throughout the majority of the recordings, to keep the stimulation paradigm conditions comparable throughout 1 h of anesthesia, and between anesthetic regimes. Previous findings in acute recordings show that the spatial frequency eliciting the most responses from neurons in the rat visual cortex is 0.08 c/d and the optimal response for temporal frequency has been found to be between 3.44 and 6.88 Hz (Girman et al., 1999). The spatial and temporal specificity was set to 0.08 c/d and 4 Hz, respectively, for the majority of the experiments (*n* = 205). The measures of spatial and temporal frequencies were performed on a separate population of units (*n* = 68). For testing of spatial frequencies the temporal frequency was kept fixed at 4 Hz while the spatial frequencies used were 0.02, 0.04, 0.08, 0.16, and 0.3 c/d. When testing the temporal frequencies the spatial frequency was kept fixed at 0.08 c/d while the temporal frequencies presented were 2, 4, and 8 Hz. The order of the frequency presentations were randomly shifted between experiments. And all visual frequencies were tested twice per state to minimize individual trial variations. Although the movement of the rat allowed its specific spatial frequency to vary between 0.04 and 0.12 c/d, only sessions where the animal was sessile throughout the whole trial were used. In other words, no spatial frequency variance occurred within one trial. Furthermore, the maximum spatial frequency change possible was calculated and found to be limited to one spatial frequency group (e.g., 0.02–0.04 or 0.04–0.08 c/d). Finally, the spatial frequency extremes such as low versus high frequencies remain unaffected by the impact of any potential movement.

### Data analysis

**Figure 1.**
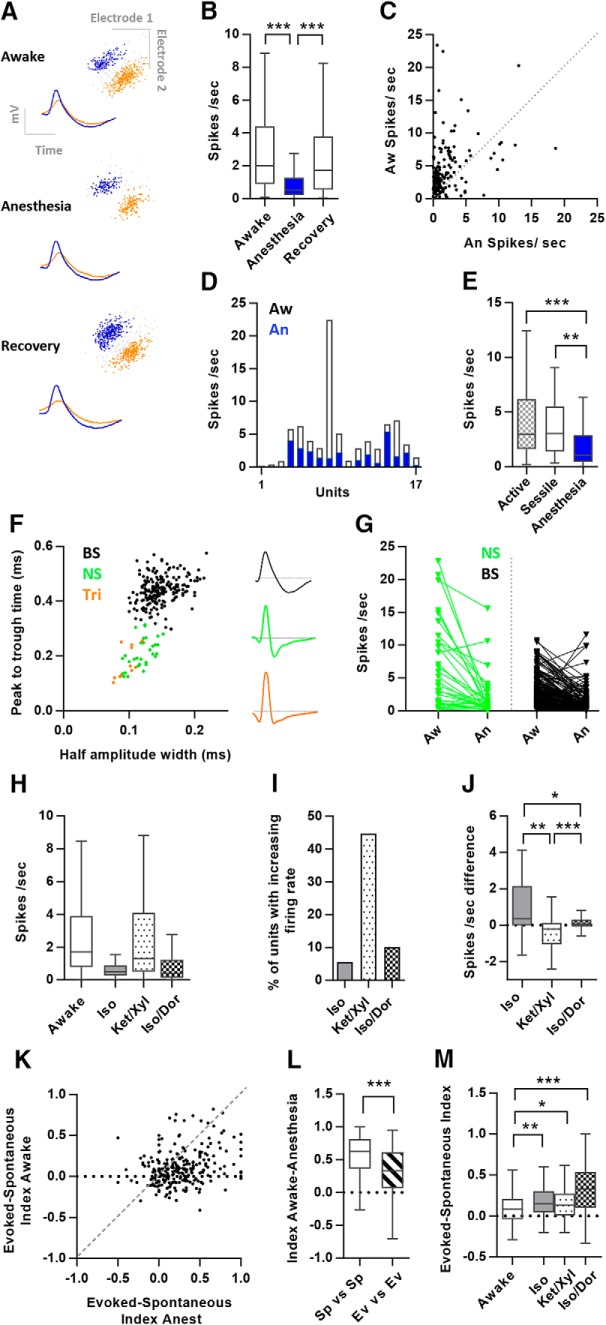
Single unit activity followed between the awake state, anesthesia, and the recovery from anesthesia. ***A***, Two example units in all three states, spike waveforms and spike clusters are shown. ***B***, Box plot of the firing rates of the units from recording sessions in the awake state, anesthesia, and recovery from anesthesia (*n* = 193). ***C***, Scatter plot of the firing rates for all single units in the awake and anesthetic state. ***D***, Single ensemble of 17 simultaneously recorded units illustrating within-ensemble variations of firing rate reduction with anesthesia (Isoflurane). ***E***, Firing rates in active and sessile sessions relative to anesthesia (*n* = 51). ***F***, right panel, Scatter plot of wave form properties of the spikes of all units, *y*-axis represent the time from peak to trough (ms) and the *x*-axis shows the duration (ms) of the peak at half amplitude. Green: NS = narrow spiking units (*n* = 31); black: BS = broad spiking units (*n* = 209); orange: Tri = triphasic units (*n* = 11). Left panel, Example waveforms of BS, NS, and Tri-units. ***G***, Firing rate of narrow spiking units (*n* = 31) versus broad spiking units (*n* = 209) in the awake and anesthetized state. ***H***, Firing rates in three different anesthetic regimes: Isoflurane/Dormicum (*n* = 145), Isoflurane (*n* = 70), Ketamine/Xylazine (*n* = 49). ***I***, Percentage of units that respond to anesthesia with an increase in firing rate. ***J***, Difference in firing rates between the first 10 and the last 60 min of anesthesia (Iso/Dor *n* = 109, Iso *n* = 65, Ket/Xyl *n* = 45). ***K***, Raster plot of the evoked- spontaneous index for each unit in the awake and anesthetic state (*n* = 257). ***L***, Box plot of indexes calculated on spontaneous activity in both states, and evoked activity in both states for each unit. ***M***, Evoked and spontaneous index for units across three anesthetic regimes (Iso *n* = 70, Ket/Xyl *n* = 48, Iso/Dor *n* = 133). All box and whiskers plots line show median, upper quartile, lower quartile and whiskers indicate Tukey interquartile range.

All analyses were, unless stated otherwise, conducted on averages of two recordings performed after 30 and 40 min of anesthesia to minimize influence of the fluctuating effect of anesthetic induction. The calculation of overall firing rates was performed as a general measure including both spontaneous and evoked time periods. The separation of the putative interneuron pool followed the same procedure as in Barthó et al. (2004) where units were classified according to their wave form properties. Two parameters were used to isolate the putative interneurons (narrow spiking) from the putative excitatory (broad-spiking) neurons: the relative time from trough to peak in ms and the width of the wave form at half amplitude of the peak. These parameters were found by Barthó et al. (2004) to produce a reliable separation of the cell classes. Fitting the data with two 2-D Gaussians resulted in a bimodal clustering of units (Fig. 1*F*). To ensure that other cell classes did not interfere with the classification of units, the following units were excluded: units that reached signal saturation, that had a low signal-to-noise ratio (SNR <2.75; Suner et al., 2005; Smith and Kohn, 2008), or units that showed the triphasic wave form typically associated with axonal activity (Robbins et al., 2013).

Evoked and spontaneous firing rates were investigated by creating peristimulus time histograms (PSTHs) of the combined sessions (repeating and alternating drifting grating and blank screens) and spike trains were convolved with a Gaussian kernel of 10-ms width and a sampling period of 1 ms. Evoked rates was calculated as the maximum firing rate of each unit across all bins following stimulus presentation, and the spontaneous maximum rates were calculated as the maximum firing rate of each unit across all blank screen bins. The first 200 ms of the spontaneous period was extracted from this maximum spontaneous rate estimation to exclude cases where units responded to stimulus OFF from the previous drifting gratings presentation. The relationship between evoked and spontaneous firing rates were quantified by an index calculated as (R1 − R2)/(R1 + R2), where R1 is the maximum evoked rate and R2 the maximum spontaneous rate. For analysis of the contribution of spontaneous versus evoked activity to this index, another index was calculated separately on spontaneous activity in both states and evoked activity in both states for each unit [(R1 − R2)/(R1 + R2), spontaneous awake (R1) and spontaneous anesthesia (R2), or evoked awake (R1) and evoked anesthesia (R2)].

The LFP trace was investigated by extracting the time period following each stimulus onset and identifying the latency to each peak and trough in the trace signature. Two response latencies for LFP were calculated as the time from stimulus onset to the first peak and trough above 1.5 * SD from the mean of the LFP trace. LFP latencies were calculated per tetrode depth and not per session to avoid errors from oversampling the same position.

Latency from stimulus onset to the peak response rate was investigated by performing Peak detection on the PSTH by calculating the peaks with firing rates that exceeded 1 SD from the mean firing rate. If several peaks after stimulus onset were present, the first peak was selected. To avoid the erroneous detection of random peaks the peak spike rate density needed to exceed 1 Hz to remove units with low firing rates. To ensure that only visually evoked units were included in the latency calculations, units were excluded if the mean evoked firing rate (±SD) did not exceed the mean (±SD) for the spontaneous time period. Additionally, a normalized firing rate was calculated per bin per unit, where firing rates of each bin was normalized to the mean firing rate (R2) of each unit (R1) (R1 − R2)/(R1 + R2). To quantify peak latency onset, the time point where firing rate exceeded 10% of the baseline to peak range was identified and kept if it remained higher than the baseline for a minimum of 25 ms. Baseline was estimated as the mean response −50 to 50 ms to stimulus onset (Brincat and Connor, 2006).

Pearson correlation coefficients (CCs) between pairs of cells were calculated in bin sizes of 10 ms, and were performed between units from a local population, i.e., from one microdrive in one hemisphere separately. Pair-wise correlations were calculated separately for the evoked and spontaneous time period, again the first 200 ms of the spontaneous time period was removed.

The presence of burst suppression in each session of anesthesia was determined by visual inspection of the LFP trace and time-frequency wavelet analyses (Tallon-Baudry et al., 1997). To be included in the pool of burst suppression sessions the LFP trace needed to have clearly visible bands of activity with high amplitude over a large frequency spectrum with clear, long-lasting isoelectric periods in between (Swank and Watson, 1949; Ferron et al., 2009).

For the analysis of temporal sequences of population activity (Luczak et al., 2009), a bin size 10 ms was used for a time period of 200 ms after stimulus onset. Varying bin size did not affect the conclusions. The details of the method are described in the results section.

To quantify the response derived from the visual stimulus with varied spatial and temporal frequencies we used two techniques. First, in accordance with Niell and Stryker (2008) and Girman et al. (1999), we selected the spatial and temporal frequency with the maximum firing rate for each unit in awake and anesthesia. Second, we computed a normalized firing rate by scaling each unit response(R) in all presented frequencies between one and zero; for this, we used (maxR-R)/(maxR-minR) per unit.

### Statistical analysis

All comparisons were checked for normality with the D’Agostino-Pearson omnibus test and Levenes test for equal variances was performed on the between-groups comparisons; *t* tests and ANOVA were used for the normally distributed data. Mann–Whitney, Wilcoxon rank-sum, Friedman repeated measures, or Kruskal–Wallis tests were performed for non-normal distributions. Dunn’s *post hoc* tests were used for Friedmann and Kruskall Wallis multiple comparisons, while Holm-Sidaks test was used for ANOVA. Box plots show median, upper quartile, lower quartile, and whiskers indicate Tukey interquartile range. All statistical tests used are included in Table 2.

**Table 1.** Parameter values for simulations

Parameter name	Awake	Anesthetized
Synaptic delay (D)	1.5 ms	
Membrane capacitance (C)	281 pF	
Membrane time constant (τ_m_)	281/20 ms	
Threshold (θ)	−50.5 mV	
Refractory period (τ_rp_)	2 ms	
Equilibrium potential (E_L_)	−60 mV	−65 mV
Reset potential (V_r_)	−60 mV	−65 mV
Excitatory synaptic efficacy (J_E_)	0.5 mV	
Relative inhibition (g)	10.5	11
Excitatory connectivity (ε_E_)	0.1	
Inhibitory connectivity (ε_I_)	0.1	
Number of neurons (N)	10000	
Excitatory neurons (N_E_)	8000	
Cortical input rate (υ_E,I,ext_)	0.7 Hz	
Cortical connectivity (ε)	0.1	
Thalamic rate (υ_E,I,pulse_)	1 Hz	
Thalamic connectivity (ε)	0.1	
Thalamic duration	50 ms	
Trials of thalamic input	100	
Simulation time	100 * 1050 ms	

### Computational modeling

Neuronal populations in primary visual cortex were represented by a randomly connected network of leaky integrate and fire neurons similarly as in Brunel (2000) with parameters given in Table 1. Cortical input was modeled as Poisson-distributed spikes depolarizing the entire network acting as an excitatory drive. Thalamic input was modeled as Poisson-distributed bursts depolarizing the entire population of inhibitory and excitatory neurons. Strong inhibitory synaptic efficacies were introduced to reproduce the low firing rates found experimentally. To simulate the transition to an anesthetized state we (1) reduced the equilibrium potential of every neuron and (2) increased the inhibitory synaptic efficacy. To investigate how the simulation results were dependent on synaptic efficacy distributions, additional numerical experiments were performed with lognormal distributed synaptic efficacies as defined in NEST (nest-simulator.org; Gewaltig and Diesmann, 2007). These simulations were performed with the same parameter values as the Brunel-type network, where the synaptic efficacies given in Table 1 represents the mean of the lognormal distribution.

**Table 2.** Statistical description of all tests

Comparison	Data structure	Type of test	Power or confidence interval (lower/upper 95%)
Figure 1*B*, awake-anesthesia-recovery	Non-normal	Friedman’s and Dunn’s *post hoc*	Awake CI: 2.74/3.83.Anesthesia CI: 0.90/1.41Recovery CI: 2.41/3.54
Figure 1*E*, active-sessile-anesthesia	Non-normal	Friedman’s and Dunn’s *post hoc*	Active CI:3.32/5.39Sessile CI:2.93/4.89Anesthesia CI: 1.48/3.47
Figure 1*G*, narrow spiking firing rates awake-anesthesia	Non-normal	Wilcoxon signed ranks test	Awake CI: 4.38/9.21Anesthesia CI: 0.81/3.29
Figure 1*G*, broad spiking firing rates awake-anesthesia	Non-normal	Wilcoxon signed ranks test	Awake CI: 2.23/2.88Anesthesia CI: 0.85/1.29
Figure 1*G*, NS aw-an difference versus BS aw-an difference	Non-normal	Mann–Whitney *U* test	BS diff CI: 1.16/1.81NS diff CI: 2.54/6.95
Figure 1*H*, awake-Isoflurane	Non-normal	Wilcoxon signed ranks test	Awake CI: 2.92/4.72Iso CI: 0.60/1.41
Figure 1*H*, awake-Isoflurane/Dormicum	Non-normal	Wilcoxon signed ranks test	Awake CI: 2.51/3.30Iso/Dor CI: 0.74/1.28
Figure 1*H*, awake-Ketamine/Xylazine	Non-normal	Wilcoxon signed ranks test	Awake CI: 1.78/3.27Ket/Xyl CI: 1.75/4.02
Figure 1*H*, aw-an difference between anesthetics	Non-normal	Kruskal-Wallis and Dunn’s *post hoc*	Iso/Dor diff CI: 1.26/2.18Iso diff CI: 1.95/3.68Ket/Xyl diff CI: −1.16/0.44
Figure 1*J*, firing rate stability over 1 h, between anesthetics	Non-normal	Kruskal-Wallis and Dunn’s *post hoc*	Iso/Dor diff CI: -0.08/0.38Iso diff CI: 0.95/2.66Ket/Xyl diff CI: −1.00/ −0.08
Figure 1*K*, index of spontaneous and evoked activity compared between awake and anesthesia	Normal	Paired *t* test	Ev/sp index awake-ev/sp index anest CI: −0.20/ −0.12
Figure 1*M*, evoked-spontaneous index change between awake and the different anesthetics	Iso/Dor diffs: Non-normalIso diffs: NormalKet/Xyl diffs: Normal	Iso/Dor: WilcoxonIso and Ket/Xyl: paired *t* test	Iso/Dor diff CI: −0.29/−0.17Iso diff CI: −0.18/ −0.05Ket/Xyl diff CI: −0.14/0.01
Figure 1*L*, comparison of spontaneous aw/spontaneous an index with evoked aw/evoked an index	Non-normal	Wilcoxon signed ranks test	Spont Aw/Spont An index CI: 0.49/0.59Evoked Aw/Evoked An index CI: 0.27/0.36
Evoked/spontaneous index comparison between moving, sessile, and anesthesia	Non-normal	Friedman’s and Dunn’s *post hoc*	Mov-Sess ev/sp CI: −0.03/0.08Mov-Anest ev/sp CI: −0.20/ −0.05Sess-Anest ev/sp CI: −0.24/ −0.06
Figure 2*B*, visually evoked trough latency (LFP)	Non-normal	Wilcoxon signed ranks test	Aw Trough CI: 0.13/0.14An Trough CI: 0.14/0.17
Figure 2*B*, visually evoked peak latency (LFP)	Non-normal	Wilcoxon signed ranks test	Aw peak CI: 0.19/0.24An peak CI: 0.28/0.42
Figure 2*C*, comparison of the difference between trough and peak latency (LFP) in awake and anesthesia	Non-normal	Wilcoxon signed ranks test	Aw trough-peak CI: 0.05/0.10An trough-peak CI: 0.15/0.26
Figure 2*D*, peak amplitude awake-anesthesia	Non-normal	Wilcoxon signed ranks test	Aw peak amp CI: 102/183.5An peak amp CI: 34/73.37
Figure 2*J*, first evoked peak latency for single units, comparison aw-an	Non-normal	Wilcoxon signed ranks test	Aw peak CI: 96.69/112.5An peak CI:141.4/164.7
Figure 2*J*, first evoked peak latency onset for single units, comparison aw-an	Non-normal	Wilcoxon signed ranks test	Aw peak CI: 63.34/87.66An peak CI:93.25/116.6
Figure 2*I*, correlation latency aw-an	Non-normal	Spearman’s	Latency diff aw-an CI: 35/61.86
Figure 2*L*, latency anesthetic regimes; comparison of aw-an	Iso/Dor and Iso: non-normalKet/Xyl: normal	Iso/Dor and Iso: Wilcoxon signed ranks testKet/Xyl: paired *t* test	Diff aw-IsoDor CI: 54.02/92.27Diff aw-Iso CI: −3.15/46.93Diff aw-KetXyl CI: −4.13/45.32
Figure 2*L*, latency anesthetic regimes; comparison between regimes	Non-normal	Kruskal-Wallis and Dunn’s *post hoc*	Diff aw-IsoDor CI: 54.02/92.27Diff aw-Iso CI: −3.15/46.93Diff aw-KetXyl CI: −4.13/45.32
Figure 3*B*, pair-wise CCs aw-an-re	Non-normal	Friedman’s and Dunn’s *post hoc*	CC Aw CI: 0.008/0.012CC An CI:0.027/0.034CC Re CI: 0.004/0.008
Figure 3*C*, pair-wise CC aw-an correlation	Non-normal	Spearman’s rank correlation	Aw CI: 0.008/0.011An CI: 0.023/0.028
Figure 3*B*, pair-wise CC aw-an-re1	Non-normal	Friedman’s and Dunn’s *post hoc*	CC Aw CI: 0.008/0.013CC An CI: 0.029/0.036CC Re1 CI: 0.004/0.008
Figure 3*B*, pair-wise CC aw-an-re2	Non-normal	Friedman’s and Dunn’s *post hoc*	CC Aw CI: 0.007/0.012CC An CI: 0.027/0.035CC Re2 CI: 0.004/0.009
Figure 3*D*, pair-wise CC evoked aw-an	Non-normal	Wilcoxon signed ranks test	CC Aw Ev CI: 0.023/0.027CC An Ev CI: 0.030/0.035
Figure 3*D*, pair-wise CC Spontaneous aw-an	Non-normal	Wilcoxon signed ranks test	CC Aw Sp CI: 0.027/0.032CC An Sp CI: 0.037/0.046
Figure 3*D*, pair-wise CC awake ev-sp	Non-normal	Wilcoxon signed ranks test	CC Aw Ev CI: 0.023/0.027CC Aw Sp CI: 0.027/0.032
Figure 3*D*, pair-wise CC anesthesia ev-sp	Non-normal	Wilcoxon signed ranks test	CC An Ev CI: 0.030/0.035CC An Sp CI: 0.037/0.046
Figure 3*E*, pair-wise CC Aw-BS	Non-normal	Wilcoxon signed ranks test	CC Aw CI: 0.005/0.011CC BS CI: 0.040/0.052
Figure 3*E*, pair-wise CC Aw-non-BS	Non-normal	Wilcoxon signed ranks test	CC Aw CI: 0.008/0.016CC Non-BS CI:0.023/0.036
Figure 3*E*, pair-wise CC Diff Aw-BS vs Aw-non-BS	Non-normal	Wilcoxon signed ranks test	CC Diff Aw-BS CI: −0.043/−0.033CC Diff Aw-NonBS CI: −0.022/-0.013
Figure 3*F*, pair-wise CC across anesthetic regimes	Non-normal	Kruskal-Wallis and Dunn’s *post hoc*	CC Aw-Iso CI: −0.036/−0.027CC Aw-Ket/Xyl CI:-0.010/-0.005CC Aw Iso/Dor CI:-0.012/-0.005
Figure 3*G*, CV in units in awake and anesthesia	Non-normal	Wilcoxon signed ranks test	CV Awake CI: 1.16/1.24CV Anest CI: 1.04/1.14
Figure 3*H*, CV within Aw-Iso, Aw-Iso/Dor, Aw-Ket/Xyl	Non-normal	Wilcoxon signed ranks tests	CV Aw-iso diff CI: −0.12/0.14CV Aw-iso/dor diff CI: −0.20/−0.05CV Aw-ket/xyl diff CI: −0.35/−0.11
Figure 3*H*, CV between anesthetic regimes	Non-normal	Kruskal-Wallis and Dunn’s *post hoc*	CV Aw-iso diff CI: −0.12/0.14CV Aw-iso/dor diff CI: −0.20/−0.05CV Aw-ket/xyl diff CI: -0.35/-0.11
Figure 4*A*, MSL correlation between states	Non-normal	Spearman’s rank correlation	Aw1-Aw2 CI: 0.29/0.60An1-An2 CI: 0.32/0.64Aw-An CI: 0.10/0.48
Figure 4*B*, single trial rank correlations (CC); comparison aw-aw, aw-an, an- an	Non-normal	Kruskal-Wallis and Dunn’s *post hoc*	Aw-Aw CC CI: 0.17/0.23Aw-An CC CI: 0.06/0.13An-An CC CI: 0.18/0.28
Skewness test; *t* test to a theoretical mean of zero on single trial rank data	Normal	One-sample *t* test	Aw-Aw CI: 0.168/0.255Aw-An CI: 0.065/0.148An-An CI: 0.193/0.284
CCs for individual populations of >15 units followed between states	Non-normal	Spearman’s rank correlation	Pop1: Aw-aw CI: 0.50/0.94Aw-an CI: −0.42/0.62Pop2: Aw-aw CI: 0.44/0.90Aw-an CI: −0.34/0.62An-an CI:0.26/0.87Pop3: Aw-aw CI: −0.03/0.77Aw-an CI: −0.06/0.77An-an CI: 0.24/0.87Pop4: Aw-aw CI: 0.05/0.80Aw-an CI: 0.32/0.88An-an CI:0.25/0.86
Skewness test; *t* test to a theoretical mean of zero on single trial rank shuffled data	Normal	One-sample *t* test	Aw-Aw CI: −0.023/0.066Aw-An CI: −0.048/0.037An-An CI: −0.066/0.027
ANOVA on single trial rank corr shuffle data	Normal	One-way ANOVA	Aw-Aw CI: −0.023/0.066Aw-An CI: −0.048/0.037An-An CI: −0.066/0.027
Single trial rank correlations (CC) versus shuffled units in all comparisons	Non-normal	Wilcoxon signed ranks tests	Data Aw-aw CC CI: 0.17/0.23Shuffle Aw-aw CC CI: −0.012/0.05Data Aw-an CC CI: 0.06/0.13Shuffle Aw −an CC CI:0.00/0.07Data An-An CC CI: 0.18/0.28Shuffle An-an CC CI: −0.07/0.02
Figure 4*C* Rank by rank measure. Comparison of single population CCs between states.	Normal	ANOVA and Tukey’s *post hoc* test	CC Aw-aw CI: 0.28/0.67CC Aw-an CI: −0.41/0.38CC Aw-an CI: 0.51/0.74
Figure 4*D*, single trial rank correlations (CC) for sessile-moving vs moving-anesthesia comparison	Non-normal	Mann–Whitney *U* test	SessMov CI: 0.094/0.177MovAnest CI: 0.032/0.106
Figure 5*B*, comparison of normalized responses to various spatial frequencies	Non-normal	Wilcoxon signed ranks tests	SF 0.02 c/d Aw and An CI: 0.44/0.56 and 0.42/0.5SF 0.04 c/d Aw and An CI: 0.47/0.57 and 0.42/0.54SF 0.08 c/d Aw and An CI: 0.40/0.52 and 0.41/0.54SF 0.16 c/d Aw and An CI: 0.36/0.48 and 0.31/0.42SF 0.3 c/d Aw and An CI: 0.39/0.51 and 0.29/0.40
Figure 5*D*, correlation between awake and anesthetized normalized unit responses in units with the awake preference of low spatial frequencies	Non-normal	Spearman’s rank correlation	Low SF pref CI: 0.23/0.61
Figure 5*D*, correlation between awake and anesthetized normalized unit responses in units with the awake preference of high spatial frequencies	Non-normal	Spearman’s rank correlation	High SF pref CI: -0.31/0.15
Figure 5*H*, comparison of normalized responses to various temporal frequencies	2 Hz: Normal4 Hz: Non-normal8 Hz: Normal	Paired *t* test and Wilcoxon signed ranks tests	TF 2 Hz Aw and An CI: 0.50/0.60 and 0.45/0.56TF 4 Hz Aw and An CI: 0.41/0.54 and 0.42/0.54TF 8 Hz Aw and An CI: 0.34/0.47 and 0.34/0.48
Figure 5*G*, first peak latencies for units under different spatial frequencies	SF 0.02 vs 0.04, 0.08, 0.16 and 0.3 c/d: Non-normal, Normal, Normal and Normal.SF 0.04 vs 0.08, 0.16 and 0.3 c/d: Normal, Normal and Normal.SF 0.08 vs 0.16 and 0.3 c/d: Non-normal and NormalSF 0.16 vs 0.16 c/d: Normal	Paired *t* test and Wilcoxon signed ranks tests	SF 0.02 c/d Aw and An CI: 99.1/136.3 and 179.6/231.6SF 0.04 c/d Aw and An CI: 104.7/134.7 and 181.6/222.6SF 0.08 c/d Aw and An CI: 92.15/121 and 158.2/205.9SF 0.16 c/d Aw and An CI: 98.27/136.6 and 144.6/194.2SF 0.3 c/d Aw and An CI: 95.34/129.2 and 126.6/181.6
First peak delay between awake and anesthesia in all spatial frequencies	SF 0.02 c/d: NormalSF 0.04 c/d: Non-normalSF 0.08 c/d: Non-normalSF 0.16 c/d: Non-normalSF 0.3 c/d: Normal	Paired *t* tests and Wilcoxon signed ranks tests	SF 0.02 c/d Diff CI: 59.8/113.8SF 0.04 c/d Diff CI: 53.57/110.1SF 0.08 c/d Diff CI: 43.25/104SF 0.16 c/d Diff CI: 26.28/88.95SF 0.3 c/d Diff CI: 5.42/73.04
Figure 5*J*, first peak latencies for units under different temporal frequencies	TF 2 vs 4 and 8 Hz:Non-normal and NormalTF 4 vs 8 Hz: Normal	Paired *t* tests and Wilcoxon signed ranks test	TF 2 Hz Aw and An CI: 91.22/135.9 and 159.6/211.8TF 4 Hz Aw and An CI: 84.6/117.6 and 155.6/203.8TF 8 Hz Aw and An CI: 84.88/122.5 and 149.5/194.7
First peak delay between awake and anesthesia in all spatial frequencies	TF 2 Hz:NormalTF 4 Hz: Non-normalTF 8 Hz: Normal	Paired *t* tests and Wilcoxon signed ranks test	TF 2 Hz Diff CI: 43.59/104.4TF 4 Hz Diff CI: 41.04/92.87TF 8 Hz Diff CI: 34.64/101.9

## Results

To investigate how anesthesia affects neural activity of units in the visual cortex, we first examined the overall firing rates of units followed between the awake state, anesthesia, and after recovery from anesthesia (Fig. 1*A*). In the awake state, the average firing rate of all units was 3.28 Hz (±0.28 Hz) and decreased to 1.16 Hz (±0.13 Hz) during anesthesia (Fig. 1*B*,*C*). Within 30 min after anesthesia, the firing rates were restored to baseline levels (2.97 ± 0.28 Hz, *n* = 193; Fig. 1*B*). The anesthesia-mediated decrease in firing rate was significant for both awake versus anesthesia and anesthesia versus recovery (*p* < 0.0001, *n* = 193, Friedman test, Dunn’s *post hoc*). Most cells (220/269 units) reduced their firing rate by 50% or more in anesthesia, while a small fraction showed increased (39/269 units) or stable (10/269 units; <10% change) firing rates with anesthesia (Fig. 1*C*). Simultaneous recordings of ensembles of units revealed that neighboring neurons may respond differently to the same level of anesthesia (Fig. 1*D*). Different intraensemble responses indicate that effects of anesthesia on cortical processing are more easily identified in recordings of the same population of units across states. Sampling was conducted across all cortical layers through the incremental lowering of the tetrodes, with an estimated layer representation of 44 units from L2/3, 36 from L4, 121 from L5 and 54 from L6, and 14 unknown.

To examine if the difference in unit activity between awake and anesthesia may be related to effects of locomotion (Niell and Stryker, 2010; Keller et al., 2012; Ayaz et al., 2013), we monitored the rat’s level of activity during recording. The animal’s movement was monitored by tracking the position of the head of the rat. Recording sessions were classified as “sessile” or “active” when the path length was shorter than 5 or exceeded 100 cm/min, respectively. The firing rate was not significantly increased with movement but unit firing rates during in the awake states were both significantly different from that of anesthesia (Fig. 1*E*, *p* = 0.0046, *n* = 51, Friedman test, Dunn’s *post hoc* test). It is likely that our small recording chamber limited movement to such extent that the relation between firing rate and running speed were not evident. Still, to avoid any confounding effects of movement in the awake state, the remaining analyses on the awake state are restricted to the sessile awake state (unless specified otherwise).

### Larger impact of anesthesia on narrow spiking units compared to broad spiking units

Because of their different types of receptors, excitatory and inhibitory neurons are likely to respond differently to the anesthetics. We therefore examined how putative inhibitory and excitatory neurons were affected by anesthesia. Based on the extracellular wave form, neocortical units can be classified into a group with broader spiking waveforms and a more narrow spiking group (McCormick et al., 1985; Bruno and Simons, 2002; Barthó et al., 2004; Niell and Stryker, 2008; Iurilli et al., 2013). The narrow spiking group posesses characteristics corresponding to inhibitory interneurons (predominantly fast spiking; McCormick et al., 1985; Barthó et al., 2004), while the broad spiking group is largely dominated by excitatory neurons. Indeed, plotting the wave form properties (Barthó et al., 2004) of peak to trough time versus half-amplitude width of all recorded units revealed a bimodal distribution (Fig. 1*F*), in accordance to previous reports (Bruno and Simons, 2002; Barthó et al., 2004; Niell and Stryker, 2008; Sirota et al., 2008; Iurilli et al., 2013). Eleven units were excluded from the narrow spiking population as they showed a triphasic wave form which is likely to correspond to axonal activity (Robbins et al., 2013).

The narrow spiking units showed a reduction in average firing rate from 6.80 ± 1.18 Hz in the awake state to 2.05 ± 0.60 Hz (*n* = 31) under anesthesia. Calculating the relative change in firing rate for each neuron gives a median reduction of 67% (*p* < 0.0001, *n* = 31.Wilcoxon; Fig. 1*G*). In comparison, the reduction in firing rate for the broad spiking population showed a median of 52% (awake: 2.56 ± 0.17 Hz vs anesthesia: 1.07 ± 0.11 Hz, *p* > 0.0001, *n* = 209; Wilcoxon). The effect of anesthesia on firing rates was significantly greater in the narrow spiking population compared to the broad spiking population (awake-anesthesia difference: 4.75 ± 1.08 Hz, *n* = 31 vs 1.49 ± 0.16 Hz, *n* = 209, *p* = 0.002, Mann–Whitney; Fig. 1*G*). The larger impact of anesthesia on fast-spiking inhibitory neurons indicates specific effects on cortical processing during anesthesia.

As the quantification of firing rate reduction performed in Figure 1*B–G* was performed on all units across all three types of anesthesia as a general comparison, we further wanted to asses how the various types of anesthetics contributed to this average. The GABAergic agonistic anesthestic regimes (Isoflurane and Isoflurane/Dormicum) resulted in a decrease in firing rate (Iso *p* < 0.0001, *n* = 145; Iso/Dor *p* < 0.0001, *n* = 70; Wilcoxon) while Ketamine/Xylazine did not produce a significant decrease in firing rate versus the awake state (*p* = 0.867, *n* = 48). The largest reduction in firing rate was for units under isoflurane anesthesia and less for Isoflurane/Dormicum (Fig. 1*H*, *p* < 0.0001; Kruskal–Wallis test, Iso/Dor-Iso *p* = 0.0002, Iso/Dor-Ket/Xyl *p* < 0.0001, Iso-Ket/Xyl *p* < 0.0001, Dunn’s *post hoc* test). Furthermore, units in the Ketamine/Xylazine condition had a large percentage of units that increased their firing rate in response to anesthesia (45%; Fig. 1*I*). Finally, to test the firing rate stability of the anesthetic regimes, we compared the firing rate change throughout 1 h of anesthesia. We found that the Isoflurane/Dormicum condition had most stable response dynamics (Fig. 1*J*, *p* < 0.0001; Kruskal–Wallis test, Iso/Dor vs Iso *p* = 0.011, Iso/Dor vs Ket/Xyl *p* = 0.002, Iso vs Ket/Xyl *p* < 0.0001; Dunn’s *post hoc* test, Iso/Dor *n* = 109, Iso *n* = 65, Ket/Xyl *n* = 45). Due to this stability, we conducted our investigation of responses to spatial and temporal frequencies with Isoflurane/Dormicum.

### Spontaneous versus evoked activity

The variable effects on firing rates of individual neurons within a population suggest that information processing of incoming sensory information is affected.

We observed a collective reduction in both spontaneous and stimulus-evoked firing rates during anesthesia. To assess how spontaneous and evoked rates were affected for each unit, we calculated an evoked-spontaneous index for each state [(R1 − R2)/(R1 + R2) R1-maximum evoked rate and R2-spontaneous rate]. We found that during anesthesia the units have a higher ratio of evoked to spontaneous activity (Fig. 1*K*, *p* < 0.0001, paired *t* test, *n* = 257). Also, by calculating separate indexes on spontaneous activity between states and evoked activity between states for each unit, we found that the relative decrease in spontaneous activity is larger than the relative increase in evoked activity (Fig. 1*L*, *p* < 0.0001, *n* = 260; Wilcoxon). Thus, our results support the proposed effect from previous investigations that an effect of anesthesia on unit activity is mainly a reduction in spontaneous activity (Niell and Stryker, 2010). This was also true for the three different anesthetic regimes when comparing the evoked-spontaneous index [Iso *p* = 0.0011 [*n* = 70], Ket/Xyl *p* = 0.028 [*n* = 48], Iso/Dor *p* < 0.0001 [*n* = 133]; (Fig. 1*M*)]. Interestingly, the Ketamine/Xylazine condtion produces a change in evoked and spontaneous activity in the units without a significant firing rate depression.

To elucidate if the lower index of spontaneous to evoked rate in the awake state was merely due to movement, we compared sessions when the animal was sessile and actively moving on a subset of units. We found no difference in the evoked-spontaneous index between sessile and moving sessions [mean index moving = 0.016 (±0.024), mean index sessile = −0.012 (±0.029), mean index under anesthesia = 0.141 (±0.030), moving vs sessile *p* = 0.6177, moving vs anesthesia *p* = 0.0133, sessile vs anesthesia *p* > 0.0001. Friedman, Dunn’s *post hoc*], supporting that the observed reduction of spontaneous activity is explained by anesthesia.

### Temporal changes of unit activity in anesthesia

Temporal structure of neuronal activity is essential for cortical processing but how the timing of unit activity within an ensemble is affected by anesthesia in the visual cortex remains unresolved. We therefore examined how the temporal specificity of units is affected by the change from awake to anesthesia.

### Visually evoked latencies are delayed under anesthesia

**Figure 2.**
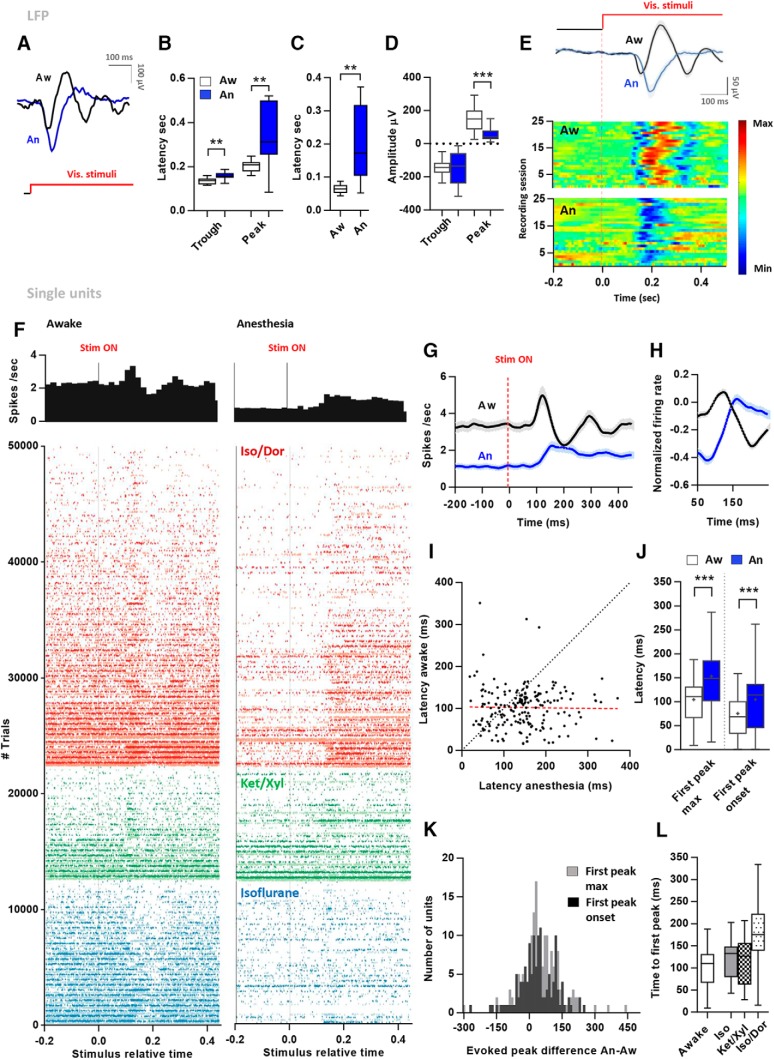
Evoked response latencies of the LFP and single units followed from the awake state to anesthesia. ***A***, Example traces from one experiment in awake and anesthesia showing the typical LFP signature following stimulus onset. ***B***, Box plot of the latency of the stimulus onset to the trough and peak of the LFP signature *1.5 SD of the mean (*n* = 17). ***C***, Comparison of the time between trough and peak in each condition. ***D***, Comparison of amplitude (mV) of troughs and peaks in each condition. ***E***, Top, Average stimulus-evoked LFP in awake and anesthesia across all experiments (*n* = 25 in nine animals). Error bars indicate SEM. Bottom, Morlet wavelet of LFP activity following visual stimulation in the awake and anesthetized animals. ***F***, Stimulus evoked firing rates (top) and raster plots (bottom) of all trials in all units in each state. One line represents one trial (96 trials per unit). Trials are ranked according to awake firing rates from high to low rate from (bottom up) for each anesthetic. ***G***, PSTH of evoked firing rates for all units followed between the awake and anesthetized state (error bars, SEM). ***H***, PSTH of normalized firing rates spanning the time period of the average peak of evoked activity. ***I***, Scatterplot showing latency (ms) to the first peak evoked response for all units in awake and anesthesia. Red dotted line indicates regression. ***J***, Box plot comparing bins of first peak max responses and first peak onset for all units between awake and anesthesia (*n* = 262,130). ***K***, Frequency distribution of the awake-anesthesia difference in latency for each unit. ***L***, Box plot showing first peak latency for units in three anesthetic regimes (Iso/Dor *n* = 133, Iso *n* = 71, Ket/Xyl *n* = 47).

Local field potentials (LFPs) provide insight into the cooperative properties of local neuronal populations and reflect synaptic activity from larger populations of neurons compared to the number of active units picked up by the electrodes (Buzsáki, 2006). To investigate the latency in response to visual stimulation, we examined the temporal profile of LFP responses to visual stimuli in the awake and anesthetized states. The average stimulus-triggered LFP signal yielded a signature with a trough followed by a peak for the majority of LFP traces investigated (Fig. 2*A*,*E*). The time to trough was significantly longer in anesthesia compared to awake (Fig. 2*B*; awake: 92 ± 14 ms, anesthesia: 112 ± 37 ms, *p* = 0.0048, *n* = 17; Wilcoxon), as well as the time to peak after trough (awake: 168 ± 49 ms, anesthesia: 307 ± 140 ms, *p* = 0.003; Wilcoxon). Interestingly, the delay between trough and peak was also different between states (Fig. 2*C*; awake: 77 ± 44 ms, anesthesia: 202 ± 111 ms, *p* = 0.002; Wilcoxon), i.e., the time to peak has a significantly larger delay than the time to the trough. Thus, the LFP signature is not only shifted in time, but appears to last longer during anesthesia. Finally, we find a large difference in amplitude of the first peak in the LFP signature (Fig. 2*D*, *p* < 0.0001; Wilcoxon). Figure 2*E* shows the average LFP trace across all experiments (top panel) as well as the response in each experimental session (bottom panel) to highlight the lack of variability between the traces. To avoid oversampling and biasing the LFP analysis, only one LFP trace per anesthesia recording session was included (17 sessions, 9 rats).

To compare how response latencies related to unit activity, we analyzed the temporal response profile of each unit. Figure 2*F* shows the spiking response of all units across all trials, as an average (top panel) and to each stimulus trial (bottom panel). To investigate the time frame of the elicited response, we computed PSTHs using a Gaussian smoothing kernel with 10-ms bins for each unit. In the awake state, the average PSTH for all units had a clear initial peak of activity with a shorter latency compared to anesthesia (Fig. 2*F*,*G*). To rule out that this pattern was not caused by the activity of a few dominating units, several tests were conducted. First, normalizing the firing rates to baseline levels showed the same tendency (Fig. 2*H*). Normalization was performed by quantifying unit firing rate (R1) of each bin to the mean firing rate of that unit (R2) (R1 − R2)/(R1 + R2). Next, we measured the time to first peak for each unit. The first peak following stimulus onset was included if the firing rate exceeded 1 SD of the mean firing rate of the unit. The time to the first peak after stimulus onset in the awake state was significantly slower during anesthesia (latency awake: 105 ± 4 ms, anesthesia: 153 ± 5 ms, *p* < 0.0001; Wilcoxon, *n* = 172; Fig. 2*I*,*J*). Following the same units from the awake to anesthetized state allowed a direct comparison of the response time in the two states (Fig. 2*I*,*J*) and revealed that most units showed a slower response to stimuli in the anesthetized state. To assess whether the delayed response also was present for the onset of the stimulus-evoked peak, we estimated the onset as the time point where firing rate exceeded 10% of baseline activity and remained above this level for at least 25 ms (Brincat and Connor, 2006). We find a significant delay of stimulus-evoked peak onset activity between the awake and anesthesia condition for our units (latency awake: 75 ± 6 ms, anesthesia: 104 ± 6 ms, *p* < 0.0001; Wilcoxon, *n* = 130; Fig. 2*J*). The mean delay between states for the first peak measure was 48 ms, with the majority of units predominantly spanning 35-62 ms (95% confidence intervals; Fig. 2*K*), while a mean delay of 30 ms was present for peak onset (spanning 14-44 ms, 95% confidence intervals; Fig. 2*K*). Furthermore, although a weak but positive correlation between latencies in the awake anesthetized states was observed (*r* = 0.16, *p* = 0.0383, *n* = 172; Fig. 2*I*) a widespread distribution of responses across populations was present (slope = 0.14, 95% confidence interval = −0.081–0.36, y intercept = 138.5, 95% confidence interval = 112.8–164.2) suggesting that latency under anesthesia was largely independent of latency in the awake state. A delay appeared to be present in all three anesthetic regimes tested; but it was only significant for two regimes: Isoflurane and Isoflurane/Dormicum [Fig. 2*L*; Iso/Dor aw-an *p* < 0.0001 (*n* = 90). Iso aw-an *p* = 0.024 (*n* = 45). Ket/Xyl aw-an *p* = 0. 10 (*n* = 37); Wilcoxon]. Furthermore, the Isoflurane/Dormicum condition had a larger delay than the two other conditions (Iso vs Iso/Dor *p* < 0.005, Iso/Dor-Ket/Xyl 0.0006, Iso vs Ket/Xyl n.s., Kruskal Wallis, Dunn’s *post hoc* test).

### Pair-wise correlations increase under anesthesia

Temporal structure of neuronal firing can be investigated by comparing correlations between spike-times of pairs of neurons. Although these correlations are often modest in magnitude, they may reflect strong constraints on information processing at the population level (Schneidman et al., 2006). It is still debated to what extent the activity of neurons in sensory cortices are correlated, with estimates ranging from 1% to 40% (Schulz and Carandini, 2010). Previous work shows that the activity of sensory neurons in layer 2/3 is more correlated during anesthesia than in the awake state (Greenberg et al., 2008). We compared correlations between unit pairs during the awake state, anesthesia, and recovery across cortical layers. Correlations were computed on pairs from the same local region (position of the tetrode wire bundle).

**Figure 3.**
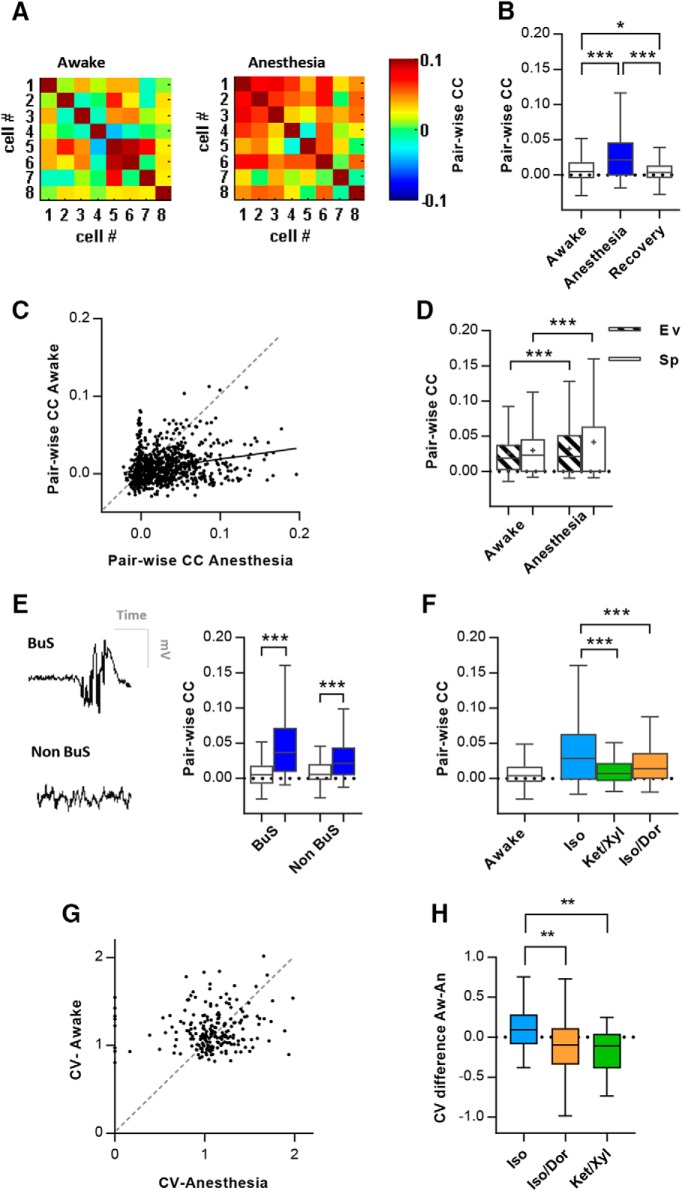
Temporal structure parameters, pairwise correlations and CV, of units followed between states. ***A***, Pair-wise CC matrix for an example population (8 units) in both states. ***B***, Box plot showing the CC for all pairwise correlations in awake, anesthesia and recovery (*n* = 733 cell-pairs). ***C***, Scatter plot of pair-wise CC for pairs of neurons in the awake and anesthetized state (*n* = 1046). Black line indicates regression. ***D***, Box plot showing the CCs during stimulus-evoked and spontaneous activity for all units. ***E***, left panel, Example LFP trace with a typical burst suppression (BuS) pattern and no burst suppression (non-BuS). Right panel, Box plot showing CCs for pairs in sessions dominated by burst suppression (*n* = 276) compared with non-BS sessions (*n* = 210). ***F***, Box plot showing the CCs for cell-pairs in three different anesthetic regimes: Isoflurane (*n* = 371), Ketamine/Xylazine (*n* = 246), and Isoflurane/Dormicum (*n* = 436). ***G***, Scatter plot of the CV for single units in the awake and anesthetized state (*n* = 218). ***H***, Box plot of the awake-anesthetized CV difference for units in each anesthetic regime (Iso *n* = 56, Iso/Dor *n* = 118, Ket/Xyl *n* = 44).

Figure 3*A* shows the correlation matrix within a population across the states. The pair-wise CC were substantially higher in the anesthetized state compared with the awake state [Fig. 3*B*; awake: 0.009 ± 0.026 (*n* = 733) vs. anesthesia: 0.026 ± 0.040 (*n* = 1053), *p* < 0.0001. Friedman, Dunn’s *post hoc*]. A small but significantly positive correlation was present between pair-wise CC in the awake state and the same cells pair-wise CC in anesthesia (Fig. 3*C*, *r* = 0.26, *p* < 0.0001, *n* = 1053, Spearman). This indicates a weak relationship between a cell-pair’s correlated activity pattern in the awake state to that same cell-pair’s activity in the anesthetized state (Fig. 3*C*, 7 outliers out of 1053 pairs were removed that exceeded 5 SD from the mean, for graphical presentation).

Interestingly, during the recovery sessions after anesthesia, the pair-wise CC were significantly lower (*p* = 0.025, Dunn’s *post hoc*) than in the initial awake sessions (recovery: 0.006 ± 0.023, *n* = 733). To examine the impact of recovery time, we separated the recovery sessions into those cases that recently recovered from the anesthetic but were apparently fully awake (recovery 1, 15-30 min after righting reflex is restored) and where the rats had recovered for 24 h (recovery 2). The correlations were significantly lower immediately after waking up from anesthesia compared with the preanesthetic awake condition (*p* = 0.0004, *n* = 651, Friedman, Dunn’s *post hoc* test). In contrast, after 24 h of recovery (recovery 2) correlations were similar to the awake state before anesthesia (*p* = 0.635, *n* = 393). This suggests a more pronounced desynchrony among cell pairs immediately following recovery from anesthesia, which is restored as the animal fully recovers from the anesthetic. This may have implications for experiments where the animal undergoes surgery on the day of recording.

Next, we investigated how pair-wise CC within stimulus-evoked and spontaneous time periods were affected by anesthesia. Our data show higher correlations in anesthesia compared to awake in both evoked and spontaneous time periods (Fig. 3*D*; evoked, *p* < 0.0001, *n* = 1039, spontaneous, *p* = 0.042, *n* = 962; Wilcoxon). Also, during spontaneous time periods, we find higher correlations compared to stimulus presentation in both the awake condition and under anesthesia (awake: *p* < 0.0001, *n* = 1049; anesthesia: *p* = 0.04, *n* = 959). Thus, the activity seems to be more correlated when the screen is blank/gray than during stimulus presentation in our population of pseudo-randomly sampled units from all layers of the neocortical column. This is in contradiction to what was reported by Hofer et al. (2011) for layer 2/3.

Burst suppression and shorter periods with an isoelectric trace in the electroencephalogram are common during Isoflurane anesthesia and have the potential to increase the correlation estimate, since units timelock their firing to bursts (UP states) (Steriade et al., 1994). We therefore tested how burst suppression during anesthesia affects synchrony among cell pairs compared with sessions not dominated by burst suppresssion in the LFP (Fig. 3*E*). As expected, periods with burst suppression showed the highest pair-wise CC (*p* < 0.0001, *n* = 276; Wilcoxon). However, sessions lacking burst suppression still showed higher pair-wise CC compared to the awake state (*p* < 0.0001, *n* = 210; Wilcoxon), suggesting that burst suppression patterns are not the sole cause of increased pair-wise correlations during anesthesia. The two comparisons were also found to be significantly different from each other (*p* < 0.0001, Mann–Whitney), indicating that burst suppression causes a greater correlation in anesthesia compared with sessions with non-burst suppressed LFP. Comparing the effect of the three different anesthetic regimes, the Isoflurane condition showed significantly higher CCs (Fig. 3*F*; Iso vs Iso/Dor *p* < 0.0001, Iso vs Ket/Xyl *p* < 0.0001, Iso/Dor-Ket/Xyl n.s.; Iso/Dor *n* = 436, Iso *n* = 371, Ket/Xyl *n* = 246; Kruskal–Wallis, Dunn’s *post hoc* test). The difference is likely due to the high prevalence of burst suppression under Isoflurane anesthesia.

The coefficient of variation (CV) was used to quantify the degree of spiking variability in single units between states. We find a higher CV in the awake state compared with CV in across all anesthetics (*p* = 0.003, *n* = 218; Wilcoxon), indicative of more regularity of the firing of a unit during anesthesia (Fig. 3*G*; five outliers that exceeded 3 SD from the mean were excluded for the graphical presentation). However, we also find differences in the impact of different anesthetics. For both Ket/Xyl and Iso/Dor, there is a significant decrease in CV with anesthesia (awake-Iso/Dor *p* = 0.002, *n* = 118, awake-Ket/Xyl *p* = 0.0002, *n* = 44); however, for Isoflurane only, there is no significant increase in CV with anesthesia (awake-Iso: *p* = 0.067, *n* = 56). When directly compared, the Isoflurane condition differs significantly from the other two anesthetics (Fig. 3*H*; Iso vs Iso/Dor *p* = 0.004, Iso vs Ket/Xyl *p* = 0.0004, Iso/Dor vs Ket/Xyl n.s.; Kruskal Wallis, Dunn’s *post hoc* test).

### Preservation of temporal sequences

Our findings of increased evoked latencies in units and LFP and changes in pair-wise correlations in response to anesthesia suggest a different temporal response profile in anesthesia compared to the awake state. In light of previous findings of preserved population-based sequences in auditory and somatosensory cortex during awake and anesthesia (Luczak et al., 2007, 2009), we examined if such temporal sequences also exist in local ensembles of the visual cortex and whether such sequences are preserved between the awake and anesthetized states.

We based our analysis on the method described by Luczak et al. (2009) with modifications to account for the reduction in firing rates, differences in evoked latencies between the states and smaller population sizes.

**Figure 4.**
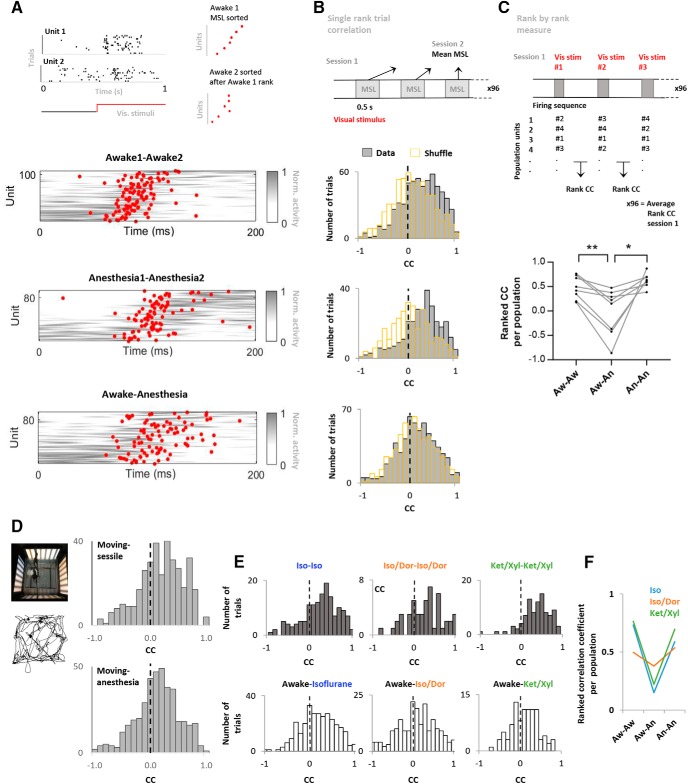
Temporal sequences within unit ensembles followed between states. ***A***, Top panel, Description of MSL measure. Left, Raster plot of two representative units firing with different MSL to visual stimuli. Right, illustration of sequence representation for MSL measure. Bottom panels, MSL (red dots) for units within ensembles, sorted by their ranked sequence (MSL) in the other session. Gray indicates activity normalized between 0 and 1 (awake-awake, *n* = 88; awake-anesthesia, *n* = 82; anesthesia-anesthesia, *n* = 78). ***B***, top panel, Illustration of the quantification of the single trial rank measure. Bottom panels, Histograms of single trial rank CCs. MSLs for individual stimuli presentations are rank correlated with the mean latency response across many stimuli presentations from a separate experimental session (awake-awake, *n* = 604; awake-anesthesia, *n* = 596; anesthesia-anesthesia, *n* = 294). Yellow outline indicates shuffled data. ***C***, top panel, The rank-by-rank measure is described. Bottom panel, Line graph showing the CCs from the rank-by-rank measure for the individual populations (*n* = 8 populations). ***D***, Single trial rank correlations for the sessile-movement (*n* = 352) and movement-anesthesia (*n* = 402) comparisons. ***E***, Single trial rank correlations for the awake-anesthesia and anesthesia-anesthesia comparisons for three different anesthetic regimes: Isoflurane, isoflurane-Dormicum, and Ketamine/Xylazine. ***F***, Example populations, from the three anesthetic regimes (Iso *n* = 15 units, Ket/Xyl *n* = 19, Iso-Dor *n* = 8).

First, we compared the sequence of mean spike latencies (MSLs) in the populations across and within states. MSL was quantified as the mean spike time of each unit during a time span of 200-ms poststimulus onset. We used this 200-ms time window since most units respond maximally during this time period, in both the awake and anesthetized condition (Fig. 2*I*,*J*). To visualize a potential sequential activation within and between states, we sorted the MSLs of one session and plotted the corresponding unit MSL in the other session. Figure 4*A* shows the MSL (red dots) and firing rates normalized to maximum and minimum in gray pseudocolor of all units, with a minimum of 9 units in each population. The preservation of sequential firing structure can be observed in all state comparisons, although the correlation is weaker comparing the awake and anesthesia sessions (awake-awake (awaw): *r* = 0.46, *p* < 0.0001, *n* = 107, anesthesia-anesthesia (anan): *r* = 0.42, *p* < 0.0001, *n* = 91, awake-anesthesia (awan): *r* = 0.30, *p* = 0.0031, *n* = 93; Spearman). To further investigate if the sequential firing structure with the MSL measure was present with individual ensembles, we analyzed individual population MSLs with a minimum of 15 units followed between states. In these ensembles we found mostly significant correlations for the awake-awake and anesthesia-anesthesia comparisons [awake-awake: mean *r* = 0.57, *p* < 0.05, four populations (one population n.s.), Spearman, *n* = 15, 19, 18, 18, anesthesia-anesthesia: mean *r* = 0.66, *p* < 0.005, three populations, *n* = 17, 17, 18]. In contrast, only one of four populations showed preserved sequence between awake and anesthesia [mean *r* = 0.37, 4 populations (n.s. except 1 population) *n* = 15, 17, 17, 18].

Second, we looked at trial correlations between the MSL for the units following a single visual stimuli presentation and the average MSL of the unit from the other state. This was performed to investigate whether ensemble responses for individual visual stimuli presentations correspond to the averages assessed in the previous MSL measure (Fig. 4*A*). For this assessment, all single rank correlations are shown that include a minimum of six units firing in response to a stimulus (Fig. 4*B*). The histograms of Figure 4*B* shows the distribution of the rank correlations in each state comparison (96 visual stimuli repetitions per averaged MSL measure). The positive skew in all single rank correlations illustrates that for the majority of stimulus presentations the firing sequence was preserved, particularly when comparing awake to awake and anesthesia to anesthesia. Furthermore, the awake and anesthesia comparisons also reveal a slightly skewed distribution of single rank correlations indicating some preservation of stimulus induced sequences across states. To assess the skewness of the distributions a *t* test to a theoretical mean of zero was performed (awaw *p* < 0.0001, awan, *p* < 0.0001, anan *p* < 0.0001, *n* = 288; *t* test). Importantly, although we find a positive skew in all state comparisons, we also find that the correlations within states are significantly different from correlations between states, i.e., the sequence preservation between awake-anesthesia is significantly less than what is present between awake-awake and anesthesia-anesthesia [*p* < 0.0001, Kruskal-Wallis; awake-awake vs awake-anesthesia (awaw-awan): *p* < 0.0001, awake-anesthesia vs anesthesia-anesthesia (awan-anan): *p* < 0.0001; Dunn’s *post hoc*; awake-awake: *n* = 604, awake-anesthesia: *n* = 596, anesthesia-anesthesia: *n* = 294].

To verify that the sequence of unit- firing was not due to systematic changes in firing rates between the states, we conducted a random shuffling of unit identity. To maintain all firing rate distributions in response to a stimulus, the random assignment of cell identity was considered to be the most robust test, i.e., shuffling unit position within a population. Figure 4*B* shows the shuffling results (yellow region) for the single trial rank. As expected, for all measures the shuffling resulted in no skewness and normal distribution of events (awaw *p* = 0.347, awan, *p* = 0.781, anan *p* = 0.411, *n* = 288; *t* test to a theoretical mean of 0), and no differences between the comparisons (*p* = 0.427, *n* = 288, one-way ANOVA).

When analyzing single rank trial correlations from awake-anesthesia to shuffled awake-anesthesia we find that the correlations (the positive skew) between awake and anesthesia is significantly different from random shuffling of unit positions (*p* = 0.011, *n* = 596). This illustrates that although the sequence preservation between awake and anesthesia is weakened, there is still some preservation intact. However, the preservation observed in awake-awake and anesthesia-anesthesia comparisons to the shuffle data are far stronger (*p* < 0.0001), suggesting that the majority of temporal sequence preservation is state dependent and show reduction between states.

Third, we implemented a new measure to reduce the impact of firing rate and mean latency on the overall scores. The rank-by-rank measure individually ranks the firing sequence of a population for each stimulus (96 repetitions) and creates an average rank score for each experimental session. This average rank score is then correlated to a corresponding measure from a separate experimental session for the same population. This way, only the relative rank position of the firing of the unit in a response to a stimulus is kept and averaged. Using this analysis we confirm that the transition between awake and anesthesia impairs the sequence preservation observed within states, i.e., CCs are significantly higher in awake-awake comparisons and anesthesia-anesthesia comparisons (Fig. 4*C*; ANOVA, *p* = 0.01; Tukey’s, awaw-awan: *p* < 0.01, awaw-anan: *p* = 0.36, awan-anan: *p* < 0.05, *n* = 8 populations). Shuffling unit identity within the ensembles removed all differences. More populations are included in this measure versus the single population-MSL correlations (first measure) since the rank-by-rank measure averages ranks across several repetitions and thus yields a more robust number across stimulus presentations, less units per ensemble is therefore required to produce meaningful data on activity within the ensemble. For populations to be included in the rank-by-rank measure more than six units in an ensemble had to be active (vs 15 units for MSL comparisons). The CCs from the rank-by-rank measure for the separate populations (Fig. 4*C*) shows that for all populations there is a lower correlation in the awake-anesthesia comparison.

Further, to show that our results were not derived from a potential variation in spatial frequency perceived by the animal between the sessile awake and anesthetized state, we quantified single stimulus rank correlations in movement sessions. During movement sessions the rat freely roamed the recording box and thereby sampling of the maximum and miniumum distance to each screen was covered throughout the experimental session. We find reduced sequence preservation in the ensembles between the wakeful moving and anesthetized state, compared with the stronger preservation between the moving and sessile session (Fig. 4*D*; sessile-moving vs moving-anesthesia: *p* = 0.0131, *n* = 352/402, Mann–Whitney, five ensembles).

Finally, to ensure that our results were not derived from the use of a single anesthetic, we tested three different anesthetic regimes. We find similar results in each regime. Similarly, the single stimulus rank correlation (Fig. 4*E*) shows a positive skew for all anesthesia-anesthesia comparisons, while awake-anesthesia has a less positive skew. Shuffling the data again removes this positive skew. Figure 4*F* shows an example population from each anesthetic regime showing the reduction in temporal sequence preservation for the rank-by-rank measure between awake and anesthesia compared with awake-awake and anesthesia-anesthesia.

### Responses between states are more similar at lower spatial frequencies

Previous studies report unit preferences to drifting gratings of particular spatial and temporal frequencies in anesthesia (Girman et al., 1999; Niell and Stryker, 2008). To date, no reports have investigated how such preferences may change for individual units from the awake to the anesthetized state. In light of the findings of changes in spike timing to visual stimulation in anesthesia, we hypothesized that an altered temporal visual processing demand could affect the unit firing responses. Changing the spatial and temporal frequencies of the stimulus were considered to represent different visual processing demands, indicating the effect of spike timing changes on visual information processing.

**Figure 5.**
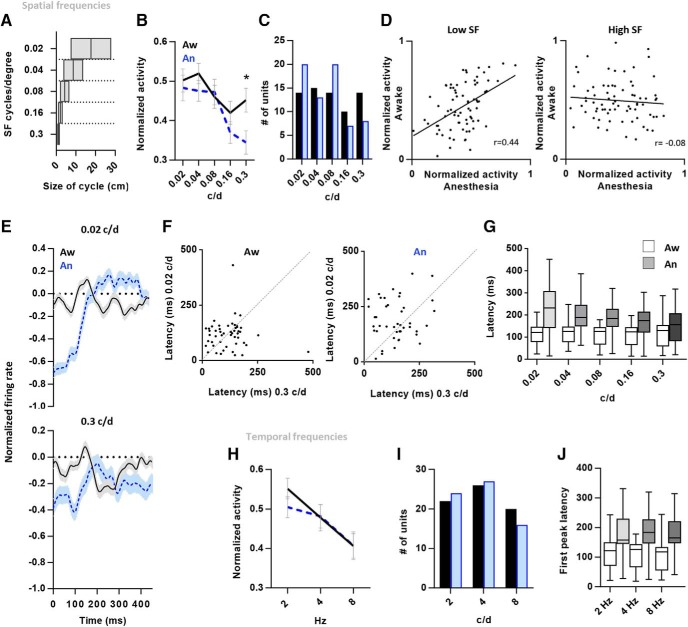
Responses to visual stimuli of different spatial frequencies between awake and anesthesia (***A*–*G***). ***A***, The possible position of the awake rats in the recording box gives a theoretical range of spatial frequencies of the stimuli. The maximum and minimum size of cycles considering varied distance to screen is plotted and show that overlap is restricted to one spatial frequency group. ***B***, Average normalized firing rates for all units in each spatial frequency (*n* = 68). Error bars indicate SEM. Firing rates normalized by scaling between 0 and 1. ***C***, Number of units responding maximally to each spatial frequency in awake and anesthesia. ***D***, left panel, Comparison of normalized activity in awake and anesthesia during visual stimuli with low spatial frequencies (0.02 and 0.04 c/d) for units preferring a low spatial frequency stimuli in the awake state (*n* = 75). Right panel, Same for units preferring high spatial frequencies of visual stimuli in awake (0.16 and 0.3 c/d; *n* = 75). ***E***, PSTH for an example unit during visual stimuli with a low spatial frequency (top panel) and a high spatial frequency (bottom panel) in the awake state and during anesthesia. ***F***, Scatterplot of evoked latencies for the lowest spatial frequency (0.02 c/d) and the highest spatial frequency (0.3 c/d) for the awake state (left panel) and during anesthesia (right panel). ***G***, Box plot of first peak latencies for units under five different spatial frequencies during awake and anesthesia. Responses to visual stimuli of different temporal frequencies between awake and anesthesia (***H*–*J***). ***H***, Average normalized firing rates for all units in each temporal frequency. ***I***, Number of units responding maximally to each temporal frequency in awake and anesthesia. ***J***, Box plot of first peak latencies for units under visual stimulation with three different temporal frequencies during awake and anesthesia. Error bars indicate SEM. Firing rates normalized by scaling between 0 and 1.

We first investigated spatial frequency responses and presented sinusoidal drifting gratings with a fixed temporal frequency (∼4 Hz reported preference for rats; Girman et al., 1999) and varied the spatial frequency. In the awake state, the animal was allowed to move in a transparent box (28 × 28 cm) surrounded by monitors presenting the visual stimulation. To calculate the maximum variation in spatial frequency introduced by the rat’s range of locations in the box, each grating cycle size was estimated at the location furthest and closest from the screen to the rat’s eyes (Fig. 5*A*). This showed that any spatial frequency response could maximally be skewed one spatial frequency group as a result of different locations of the rat in the chamber. The analyses were restricted to periods when the animals were sessile and the measurement of anesthetic responses was conducted with Isoflurane/Dormicum anesthesia due to the low changes in firing rate over time with this anesthetic (Fig. 1*K*).

Comparing the same units across states we found a general tendency across units to prefer lower spatial frequencies in anesthesia compared to the awake state (Fig. 5*B*,*C*). Specifically, preferred frequencies during anesthesia were between 0.02-0.08 c/d, like previously reported (Girman et al., 1999; Niell and Stryker, 2008), while the normalized responses to the highest spatial frequency (0.3 c/d) was significantly lower in anesthesia compared to awake (Fig. 5*B*, *p* = 0.012, *n* = 68; Wilcoxon). Furthermore, the units differed in their change of preferred spatial frequencies, where 28 units shifted toward lower, 22 units toward higher and 18 units showed no change in preferred spatial frequency. By separating and examining the units by their preferred spatial frequency in the awake state (Fig. 5*D*), we observed that units originally selective for the highest frequencies (right panel: 0.16 and 0.3 c/d) in the awake state appeared to shift their preferences toward lower (left panel: 0.02-0.04 c/d) frequencies during anesthesia. In contrast, units originally selective for the lower frequencies, showed no systematic change with anesthesia. This was true both for normalized activity during the different stimulus presentations and the units’ preferences. Interestingly, no shift was present in the units preferring 0.08 c/d. There was a significant correlation for the normalized activity of low frequency selective units between awake and anesthesia (*r* = 0.44, *p* < 0.0001, Spearman, *n* = 75; Fig. 5*D*, left panel). For the high frequency selective units we found no correlation between the preference in the two states indicating a change of preference between states toward the lower end of the spectrum (*r* = −0.08, n.s, Spearman, *n* = 75; Fig. 5*D*, right panel).

We also investigated the first peak latency of units during the different spatial and temporal frequencies (Fig. 5*G*,*J*). Interestingly, in anesthesia there appeared to be a gradually decreasing latency with each increase in spatial frequency (Fig. 5*E–G*) while the latencies were surprisingly similar in the awake state in all spatial frequencies tested. Figure 5*E* shows an example unit with a larger latency difference between awake and anesthesia in the spatial frequency 0.02 c/d compared with 0.3 c/d. Units had an overall larger latency difference between awake and anesthesia during the lowest spatial frequency (0.02 c/d) compared with the highest (0.3 c/d; Fig. 5*G*, *p* = 0.03, paired *t* test, *n* = 39). The same was true for the second lowest (0.04 c/d) versus the highest (0.3 c/d; *p* = 0.04, paired *t* test, *n* = 41). Also, the majority of units in the 0.02 versus 0.3 c/d comparison showed this change in latency (Fig. 5*F*). There was still a significant first peak delay between awake and anesthesia in all conditions (0.02 c/d: *p* < 0.0001, *n* = 50; 0.04 c/d: *p* < 0.0001, *n* = 55; 0.08 c/d: *p* < 0.0001, *n* = 48; 0.16 c/d: *p* < 0.0005, *n* = 47; 0.3 c/d: *p* = 0.019, *n* = 41; 2 Hz: *p* = 0.0003, *n* = 48; 4 Hz: *p* < 0.0001, *n* = 48; 8 Hz: *p* < 0.0001, *n* = 45; paired *t* tests). When testing the same units for visual stimuli with three temporal frequencies (2, 4, and 8 Hz), no significant differences between normalized firing rates and unit preferences was found between the awake and anesthetized state (Fig. 5*H*,*I*).

In addition, no latency differences were found between the three different temporal frequencies in either state (Fig. 5*J*), suggesting that the varying effect of anesthesia on latency with no awake change is specific to activity under differing spatial frequencies. It would appear as the processing of spatial content rather than the contents speed is affected by anesthesia. Regardless, the presence of a state-dependent latency difference, which is determined by the spatial frequency of the visual stimuli highly suggests an altered visual processing during anesthesia.

### Modeling awake-anesthesia data

Anesthetics appears to work by the direct effects on the neurons and indirect network effects due to the altered activity of neurons. In particular, several anesthetic agents seem to increase inhibition in a network. In principle, it should therefore be possible to account for the presently observed differences between anesthetized and awake brain states in a network model for visual cortex including sufficient biophysical details to mimic the effect of presence or absence of the anesthetic agent. The present type of data where the neural activity is measured for the same set of cells in both the anesthetized and awake states allows for a new set of validation tests that candidate cortical network models should pass. To illustrate this approach we next compare our experimental results with predictions from model simulations using NEST (nest-simulator.org; Gewaltig and Diesmann, 2007). Here, we consider one of the most well-established and analyzed network models mimicking cortical network activity, namely the Brunel network (Brunel, 2000) with two recurrently connected populations, one excitatory and one inhibitory, of LIFs.

As described in detail in Materials and Methods, the LIF neurons integrate synaptic input currents, and when the membrane potential reaches a preset threshold value, an action potential is added, and the membrane potential is reset to a preset reset value. Here, the neuron and network parameters were first set to give a network behavior plausibly mimicking an “awake” state, i.e., a state with asynchronous firing and firing rates in rough agreement with the present awake-state recordings. To mimic the anesthetized state of the same model network we modified the network in two ways. First the synaptic inhibition, i.e., the weight of the inhibitory synaptic current, was increased, and second the equilibrium value defined by the leak potential was lowered for all neurons in the network. The latter modification was meant to mimic an overall hyperpolarization of the neurons induced by a more global inhibitory action induced by the anesthetic agent. Most work on the Brunel model has considered homogeneous networks, i.e., the same synaptic weights between all neurons in the same population (Brunel, 2000). However, recently variants of the model including variations in the synaptic weights have been considered, and in particular a variant with lognormally distributed weights have been observed to make the dynamics more similar to experiments (Iyer et al., 2013; Teramae and Fukai, 2014; Hagen et al., 2016). Here, we considered both homogeneous and “lognormal” networks to explore how robust our model findings were to this aspect of the network connectivity.

**Figure 6.**
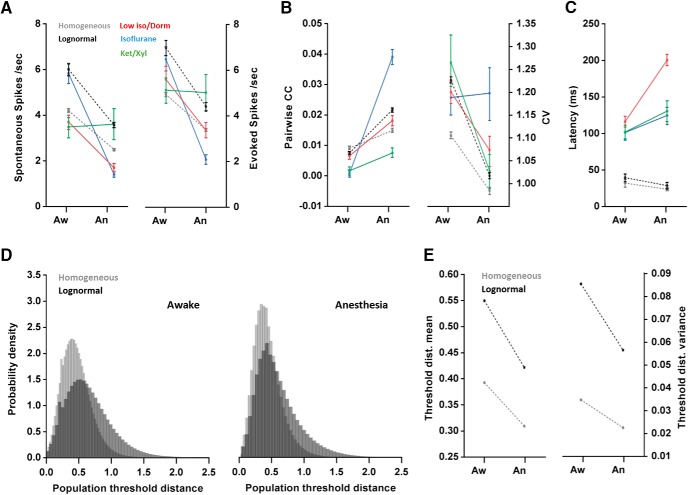
Awake-anesthesia data compared to data from the Brunel-type network model. Anesthetics are color coded. ***A***, left panel, Spontaneous rate. Right panel, Evoked rate. ***B***, left panel, Pairwise CCs. Right panel, CV. ***C***, Peak latency (ms). ***D***, Probability density of the membrane potential in the awake (left panel) and anesthetized (right panel) network model. ***E***, left panel, Network model threshold distributed mean. Right panel, Threshold distributed variance.

The simulation results were compared to the electrophysiological recordings in the two states and with different anesthetics, results are presented in Figure 6. Isoflurane-based anesthetics and the simulated data showed reduced firing in the anesthetized state, both the spontaneous rate (Fig. 6*A*, left panel) and the evoked peak rate (Fig. 6*A*, right panel). Both the homogeneous and the lognormal network showed the same trend, i.e., reduced firing rates in the anesthetized state, as the experiments for Isoflurane-based anesthetics. All three types of anesthesia were observed to increase the latency from stimulus onset to the peak response in the PSTH. This phenomenon was not captured in any of the models which both showed little variation of latency between the states (Fig. 6*C*). Note, however, that any increased latency at the geniculate level would not be captured in these models as the input to the network (mimicking input from LGN) was assumed identical in the awake and anesthetized states. However, both models captured the observed increase in pairwise CC (Fig. 6*B*, left panel). Anesthesia caused decrease in the CV in both models and the experiments, except for pure isoflurane where no change was observed (Fig. 6*B*, right panel).

Extracellular recordings cannot measure subthreshold variations of the membrane potential, but these are fully available in the models. As the models seemed to capture salient features of the differences between the awake and anesthetized states, we next investigated the models’ distributions of subthreshold membrane potentials in the different states (Fig. 6*D*). These panels show the probability density of the membrane potentials where the population threshold distance is measured as percentage of the threshold value. The key observation was that in both models the distribution is narrower and more shifted toward the firing threshold in the anesthetized state than in the awake state (Fig. 6*D*). Despite this shift of the mean of membrane-potential distribution toward threshold with anesthesia (Fig. 6*E*, left panel), the firing in the awake states was still larger. This likely reflected the larger variance (Fig. 6*E*, right panel) in the awake state, i.e., that in the model the firing was more “fluctuation driven” than “mean driven” (Renart et al., 2007; Kriener et al., 2014).

Note that we consider these model observations to be quite preliminary and maybe primarily suited to illustrate a workflow for comparing experimental data with candidate network models. A more comprehensive comparison between models and the present experiments should be considered when more detailed candidate network models of the rat visual cortex becomes available.

## Discussion

Visual cortical processing has been studied in great detail in anesthetized and awake head-restrained animals, but it still remains unclear how the temporal dynamics of neurons within an ensemble differ between states. Using chronic extracellular recordings of single units we show that simultaneously recorded units preserve a temporal sequence in response to visual stimulation. While this sequence of firing is maintained within states, it is weaker for the same ensemble across states indicating a change in temporal dynamics under anesthesia. This change is further confirmed from the slower stimulus-evoked responses during anesthesia compared to awake both on the population level (LFP) and in single unit responses and from the increase of pair-wise correlations between cell pairs. Moreover, we show that changing the spatial frequency of the visual stimuli results in a change in unit response-speed in anesthesia, while the same units remain more unaffected in the awake state. Comparing single-unit results such as these to network models is beneficial for improving models on network activity. We exemplify such a workflow by implementing a Brunel-type network model, and reproduce many features from the dataset in our model.

The findings that receptive field sizes are reduced in somatosensory cortex under anesthesia (Armstrong-James and George, 1988) while they appear to increase in the visual cortex under anesthesia (Haider et al., 2013) highlights the need for separate investigations in each cortical area to reveal the impact of anesthesia on single units. This may in part be due to, e.g., differences in compositions of neuronal receptor subtypes that would make them differentially susceptible to a particular anesthetic drug, e.g., GABA-A receptor subunit combinations vary greatly between cortical areas, and several anesthetics are known to operate on different GABA-A receptor subunits. Thus the effects of anesthesia is likely agent and area specific (Vahle-Hinz and Detsch, 2002). For this reason, we compare three different anesthetic agents to reveal the effects of anesthesia on unit activity in the visual cortex.

### Chronic recordings

Chronically implanted tetrodes may have several advantages compared to acute recordings. First, activity from the same units and LFP oscillations can be reliably followed across days enabling direct comparisons of changes to the neural population in response to manipulations. Second, recordings can be made in freely behaving animals lowering potential stress of restraint and potential impact of reduced head motility on population responses. Also, acute inflammatory responses due to electrode implantation is reduced as the animal is left to recover for several days after surgery after which the tetrodes are slowly lowered into the cortical column without reopening the implantation site. Although investigations of visually evoked processing in freely moving animals as they were conducted here, have their limitations with less control of eye movements and pupillary changes, our results show that reliable visual response patterns can be detected and that the cross-state recordings reveal properties of cell ensembles that are otherwise not possible to achieve.

### Diverse effects of anesthesia on single unit firing rates

The strong reduction in firing rates in response to anesthesia (Fig. 1) is in accordance with previous findings from the visual cortex (Villeneuve and Casanova, 2003; Schummers et al., 2008; Sleigh et al., 2009; Vizuete et al., 2012). However, following the same population of units across states revealed nonhomogenous, cell-specific and individual unit differences in response to anesthesia (Fig. 1*C*), even among neighboring neurons (Fig. 1*D*). Since each neuron possess a different composition of receptor subtypes, display variation in neuronal morphology and electrophysiological response characteristics (Edwards et al., 1990; Steriade, 2004; Van Aerde and Feldmeyer, 2015) the variations of the effect of anesthesia could arise from local factors alone. In addition, each neuron also holds a unique position in the cortical circuitry, and as such the variation observed in reponses between neighboring neurons may be due to indirect network effects. Similarly, the larger reduction in firing rates of narrow spiking (putative interneurons) units compared to the broad spiking (putative excitatory) population (Fig. 1*G*) may reflect the molecular targets of anesthesia in the local circuitry (e.g., higher GABA receptor density on parvalbumin positive inhibitory neurons; Klausberger et al., 2002) or originate in altered upstream or presynaptic activity. Regardless, the different impact would produce highly different levels of inhibition between states (Haider et al., 2013). In contrast to the our findings, some previous reports find little impact on overall V1 firing rates with anesthesia (Pisauro et al., 2013; Durand et al., 2016). This could be partly explained by the use of urethane anesthesia in these experiments, since urethane has been shown to exert differential effect on neocortical neuronal activity compared to other anesthetics (Maggi and Meli, 1986; Dyer and Rigdon, 1987).

The increased evoked-spontaneous index in anesthesia compared to awake was mostly due to reduced spontaneous activity (Fig. 1*L*). This is consistent with findings from mice comparing separate populations (Niell and Stryker, 2010). The heavy reduction in spontaneous activity may be caused by reduced top-down and feedback processing in corticocortical connections during anesthesia (Mashour, 2014; Raz et al., 2014).

### Increased evoked latency during anesthesia

The shorter stimulus-evoked latencies in the awake state compared to anesthesia in the LFP signature was reflected in the response latencies of single units (Fig. 2*B*,*I*,*J*). The slower response to visual stimuli under anesthesia has implications for visual processing and interpretation of results from studies conducted under anesthesia. Following the units across states, we found that the slower response due to anesthesia differed between units giving an unpredictable effect on network processing. Numerous reports suggest that high temporal specificity is a trait of efficient processing, and that the coding of different signals is derived from slight variations in the temporal structure (Mainen and Sejnowski, 1995; Gasparini and Magee, 2006; Tiesinga et al., 2008; Luczak et al., 2013). The increase in response delays and not least variability of delays under anesthesia will profoundly affect the temporal structure and impair the efficiency of the network such as “binding” of the sensory image during cortical processing (Engel and Singer, 2001).

The increased response latency with anesthesia is in accordance with Pisauro et al. (2013) and Durand et al. (2016), recording multiunit-activity (MUA) and single unit data from separate animals where analyses are conducted on separate pools of units. Pisauro et al. (2013) report a peak latency of ∼92 ms in awake V1 mouse in MUA recordings, and ∼189 ms in anesthesia (two regimes: urethane and Isoflurane), while Durand et al. (2016) report ∼97 ms in awake mouse V1 single units and ∼120 ms in a separate anesthesia population (urethane). These numbers are comparable to our latency measure of awake (∼105 ms) and anesthesia (∼153 ms). Our results verify these findings and elaborate by showing how individual units in a population increase their stimulus-evoked latency at a varied degree between the states for both peak rates and peak onset (Fig. 2*I*,*K*). In addition, the prolonged LFP response during anesthesia is comparable to that reported in Haider et al. (2013), where they show large differences in the dynamics of inhibition between the states.

Furthermore, we show how different anesthetics cause different unit response delays with the anesthetic regimes with a predominantly GABAergic agonistic action having the largest impact on latencies (Fig. 2*L*). Wang et al. (2014) found that a synchronized state (anesthesia) is associated with longer latency in visual cortical neurons. They argue that it is not the anesthetic state itself, but rather the synchrony that is coupled to increases in latency. However, in their study they manipulate the presence of the synchronized state with on/off usage of Isoflurane on top of a stable urethane anesthesia. In light of the present findings and others (Cheung et al., 2001; Haider et al., 2013), it could be argued that it is the choice of anesthetic (the GABAergic action of Isoflurane) that is responsible for the increases in neuronal delay and not the synchronized state. Thus, the degree of latency observed in the current study and that of Wang et al. (2014) is caused by the use of GABAergic agonistic anesthetics. A smaller delay was indeed apparent in the Ketamine/Xylazine condition.

When comparing findings from different primary cortical areas, it appears like the various cortical areas are influenced differently by anesthesia. While units in the somatosensory cortex of rabbits appear to show delays with anesthesia (Angel et al., 1973), much like our finding in the visual cortex, units in the auditory cortex appear to show the opposite result. A study by Ter-Mikaelian et al. (2007) on the Mongolian gerbil shows that the response to tones in auditory units is faster under anesthesia than in the awake state. It is plausible that the choice of a non-GABAergic anesthetic in their study was responsible for this discrepancy. A previous investigation finds larger delays in auditory anesthetic responses of cats with the GABAergic agonistic Isoflurane compared to pentobarbital anesthesia (Cheung et al., 2001). However, the reported awake response time in Ter-Mikaelian study is much longer than the anesthetic regime that gave the longest delay (Isoflurane) in the Cheung study, while the pentobarbital response was comparable between the studies. Although comparing latency estimates from different investigations may be problematic due to varying criteria of what qualifies as an evoked response, similar latency values with a non-GABAergic anesthetic in the two experiments suggests a degree of validity to the comparison. Accordingly, awake response times appear to be delayed in auditory cortex compared to several forms of anesthetics, including the anesthetic responsible for the greatest delay in all visual studies (Isoflurane). This yields support to the conclusion that both cortical area and anesthetic regime are determinants of the impact of anesthesia on the latency of units.

### Pair-wise correlations increase under anesthesia

The low pair-wise correlations of neurons in the sensory cortices may indicate the fraction of shared inputs (Alonso et al., 2001; Ko et al., 2011) rather than the direct connectivity between neurons (Moore et al., 1970). The increased pair-wise correlations in anesthesia compared with awake (Fig. 3) in our data are in accordance with recent findings that correlations may vary according to state of the animal (Greenberg et al., 2008; Ecker et al., 2010; Renart et al., 2010), suggesting that the structure of population activity depends on brain state. We supplement these findings by adding that the increase of pair-wise correlations occurs both under stimulus-evoked and spontaneous events. This suggests that the synchronization induced by anesthesia occurs regardless of changing visual parameters and could likely reflect a general up-regulating mechanism. This is in agreement with what has been reported on a larger scale across brain regions using fMRI and LFP (Bettinardi et al., 2015). We also find that spontaneous time periods in both states induce a higher pair-wise correlation between the units than during evoked, which implies that visual stimuli appear to desynchronize the activity of unit pairs, irrespective of state. A similar tendency for higher pair-wise correlations during spontaneous time periods versus evoked has been reported in the anesthetized monkey (Smith and Kohn, 2008). Furthermore, although a previous study describes a reduction in neuronal variability with sensory stimulation (Churchland et al., 2010), this comparison is across stimulus trials and does not reflect the correlation between units in a single trial. The result of Churchland et al. (2010) is therefore more applicable to our findings on the stability of visually evoked temporal sequences within states.

Furthermore, while pair-wise correlations returned to baseline levels 24 h after anesthesia, the correlations were significantly lower 30 min after animals recovered from anesthesia than the preanesthetic awake condition. This could indicate some desynchronizing mechanism during the recovery from anesthesia. Future investigations are needed to reveal the time course and synchrony of the recovery phase.

### Temporal sequences within and between states

In several sensory systems, short-latency responses correlate with simple stimulus features, whereas later responses often represent more complex features. Our recordings from the same local populations in awake and anesthesia enabled direct comparisons of sequence in firing activity within a local population and across states. We first showed that within populations of recorded units in the visual cortex there is a clear temporal sequence of firing during specific visual stimulation that is preserved between awake recording sessions (Fig. 4). Moreover, we find a clear temporal structure within the populations in response to visual stimuli when comparing separate time points of anesthesia. Thus, units in the visual cortex appear to share the trait discovered by Luczak et al. (2007, 2009) from the auditory and somatosensory cortex. They reported that a sequence structure in firing activity within a population is retained across types of stimuli and occurs spontaneously during UP states, suggesting a stereotyped mode of information flow in a local cortical population (Luczak et al., 2007, 2009). They suggested that neurons firing earliest in the sequence would reflect an initial processing of incoming information; neurons firing at later time points would have access to the results of computations made by earlier firing ones and, thus, be capable of more sophisticated analysis.

While previous investigations were limited to comparing separate populations, we show that the temporal sequence of firing within a population is reduced in the transition between awake to anesthesia. We find this both in overall MSLs per unit and in the correlations of ranks between populations of units. This suggests a state-dependent preservation of temporal firing structure within a population where the cortical state determines the local dynamics that produces the temporal firing sequence within the cortical microcircuit. This suggests less stereotypic temporal structure in V1 than previously suggested from findings in auditory and somatosensory cortex (Luczak et al., 2009). However, the partly preserved sequence of activity between awake and anesthesia suggests that some connections may be more hard-wired and independent of state.

### Increased delay with lower spatial frequencies in anesthesia

We observed a preference toward higher spatial frequencies of V1 neurons in the awake state compared to anesthesia. This is accordance with preferred spatial frequencies during anesthesia in anesthetized rodents (Girman et al., 1999; Niell and Stryker, 2008; Gao et al., 2010) and an increases in spatial frequency preferences in awake mouse V1 (Andermann et al., 2011; Durand et al., 2016) and in awake versus anesthetized primate LGN (Alitto et al., 2011). However, while previous investigations compared responses in separate experiments, our chronic recordings allowed the detection of changes in single units recorded under anesthesia and in the awake state.

We find that for the highest spatial frequencies (0.3 c/d), it is mostly units in the awake state that respond (Fig. 5*B*,*C*). When separating units on their preference toward low or high frequencies in the awake state, we found that the low preferring units remained mostly unchanged under anesthesia, while the high frequency group shifted their preference toward lower spatial frequencies in anesthesia (Fig. 5*D*). This shows that it is specifically units with high spatial frequency preference that change with the transition between awake to anesthesia and is not a result of a collective shift in all units toward lower preferences. High spatial frequencies may demand high temporal specificity and perhaps the prolonged evoked latencies and disrupted firing sequences in anesthesia impair the efficiency of processing higher frequencies.

Finally, we show that changing the spatial frequency of the visual stimulus induces differing response-latencies in anesthesia and not in the awake state (Fig. 5*G*). Visual stimuli consisting of drifting gratings with a broader width (low spatial frequency) produces more prolonged latencies in anesthesia compared to stimuli with a more narrow width (high spatial frequency). Such a change may be expected due to the slower increase of contrast in each unit’s receptive field during low spatial frequency conditions. However, the absence of this change in the awake states suggest a difference in the processing of these stimuli in the two states. The unit latency under all spatial frequencies in the awake state is surprisingly similar. In light of the findings by Haider et al. (2013) of increased and faster inhibition in the awake state, it is possible that fast inhibition terminates the more prolonged responses found in anesthesia under lower spatial frequencies. Thus, if responses are inhibited and terminated in the awake state, this may conceal the response differences found in anesthesia. The finding that awake inhibition dominates sensory processing while GABAergic agonistic anesthesias induce delays and alterations in temporal responses may be counterintuitive. However, since we also find that putative fast-spiking interneurons have a larger firing rate decrease compared to the general population, this suggests that their ability to regulate/inhibit activity is diminished. Fast spiking inhibitory neurons indeed have a higher GABAergic receptor density (Klausberger et al., 2002), allowing a direct agonistic action from the anesthetics. It can be hypothesized that it is the potentially direct influence of anesthetics on fast-spiking inhibitory neurons that remove the large and fast inhibition of responses to the varying spatial frequencies that is present in the awake state.

In addition, we observe with interest that many of salient features of the observed differences between awake and anesthetized states are reproduced qualitatively by the relatively simple Brunel-type model networks when the anesthetic actions are assumed to increase inhibition in the network (Fig. 6). In systems neuroscience such a model-based analysis approach where candidate network models are compared quantitatively with experimental data, is still in its infancy, primarily due to the lack of candidate network models amenable for such analysis (but see Potjans and Diesmann, 2014; Hagen et al., 2016). As more such models become available, we expect the present workflow for comparing experiments with models to become more prevalent.

## Synthesis

Reviewing Editor: Tatyana Sharpee, The Salk Institute for Biological Studies Decisions are customarily a result of the Reviewing Editor and the peer reviewers coming together and discussing their recommendations until a consensus is reached. When revisions are invited, a fact-based synthesis statement explaining their decision and outlining what is needed to prepare a revision will be listed below. The following reviewer(s) agreed to reveal their identity: Davide Zoccolan

The study reports a set of very systematic and comprehensive measurements of the impact of different kinds of anesthetics on the activity of different cell types in the brain. The results will have important impact on the field. However, both reviewers felt strongly, and I agree, that the presentation needed substantial improvements including figure re-arrangements. The sections that were especially not clear are on rank correlations. The detailed comments are below. Also, some discussion is needed as to the choice of particular anesthetics used, and some additional detail to characterized the “sessile” state. Further, the work by Churchland et al. Nature Neuroscience 2010 is relevant to results described on lines 425-432.

Reviewer 1:

In this study, the authors compare some neuronal response properties of rat V1 neurons in the anesthetized and awake state. These properties include magnitude of the spontaneous and evoked responses, latency of the response and the temporal pattern of firing of ensembles of simultaneously recorded neurons. The main strength of the study is the fact that it based on chronic recordings from implanted animals, which were tested in either the awake or anesthetized state, thus allowing probing the same neurons under both states. In addition three different kinds of anesthetics were used. On the other hand, the main weakness of the study is that the writing is quite poor, especially in the Results section, where not only analyses and results are described approximately, but the material presented in the figures is not described in sequential order: the authors jump back and forth among different panels of a figure or even among different figures, sometime mislabeling the figures they refer to in the main text. This has made the Results extremely difficult and tiring to read, with the consequence of lowering considerably the understanding of the authors' conclusions. As a consequence, I found myself unable to fully gauge the solidity and originality of the findings reported by the authors. On top of that, I also found that the discussion of similar results form previous studies is too slim and does not quite cover the existing literature.

In any event, given that the experiments the authors carried out seem to be solid enough (although not perfect in their design: see the lack of tracking of head direction in the awake recordings), and given the fact that the topic is of some interest (not many papers have followed the same neurons across awake and anesthetized states), I think that the manuscript deserve the chance to be revised and reconsidered. But this revision needs be very deep and careful: 1) the order of the figures should be totally revised, making it sequential and aligned to the description provided in the text; 2) the metrics used in the analysis should be completely described in the Methods (it is not enough to refer to previous studies that have used them) and also described again (shortly) in the Results, in the first instance that they are used; 3) and the whole text should be revised by some colleague of the authors, possibly mother-tongue, that should critically read the full manuscript and honestly point to the authors what she/he does and does not understand. Only after that this is done, and the manuscript has been considerably improved, it will be possible to truly assess the solidity and impact of the manuscript.

Below are my specific comments.

1. Introduction and Discussion: the recent article of Durand et al (J Nerosci, 2016) should be mentioned somewhere in the introduction and then discussed at length in the Discussion, since that articles address many of the same questions investigated by the authors. The authors should compare the two studies and explain what similarities and differences are found across them.

2. Lines 210-211: “Evoked firing rates were calculated as the maximum firing rate of each unit across all bins following stimulus presentation”. What bins are the authors referring to here? I guess they binned the time axis and computed the average firing rate (FR) in consecutive time bins, but it is not mentioned the width of these bins; neither that the time axis has been binned. The authors cannot simply assume that the reader will know what they are talking about. They need to provide all the details of their methodological approach, and possibly in a sequential, logical order.

3. It was not clear at which cortical depth the presented data were collected. The authors state in the methods that tetrodes were implanted “into the primary visual cortex, about 300 um below the dura mater at an angle of 30-40 degrees in the lateral to medial direction” (L102). Then they point out that the electrodes could be easily moved up and down and that “the recording location was extrapolated from deepest trace identified by histological inspection of the sections and the tetrode-tuning log” (L 157-168). Thus is not clear whether the recorded neurons are all from the same layer, or are presented independently from their depth. From what I understood, most likely recordings come from layer 2/3. This issue should be explained by the authors as, especially in the anesthesia state, this could affect the results. More importantly, if the authors sampled multiple layers, they should try to segregate their data also on a layer-by-layer fashion. If they cannot do so, they should explain why.

4. In the methods, the author state that the Isoflurane concentration was set at 1.5% for the condition 'Isoflurane only“ because this was the ”minimum alveolar concentration for adult rats (Mazze et al. 1985), while for the Isoflurane/Dormicum “conditions were initially tested with pilot studies to achieve the lightest possible level of anesthesia” (starting from line 120, to line 130). As also the authors seem to be aware of (because they adjust the concentrations), the correspondence between depth of anesthesia and % of isoflurane varies a lot across animals, and across studies in the literature. For example, 1.5% is reported as “light anesthesia” in Hudetz et al 2003, while is defined as “deep” in Silva et al 2010 (Silva and colleagues define light anesthesia at 0.8% Isoflurane). I would specify this in the methods and report a wider range of literature, with some more recent study.

5. The author reported the “low signal-to noise ratio” as a method for units exclusion from the analysis (L 208). This should be specified better. What was the threshold for exclusion? The SNR was calculated on spontaneous or stimuli-evoked activity?

6. The units selection reported at line 229-232 is the same of point 4? Please clarify

7. Lines 214-215: “The ratio between maximum evoked rate (R1) and spontaneous rate (R2) was calculated as calculated as (R1-R2)/(R1+R2) for each unit”. This should not be called a ratio. It is confusing. A ratio would be R1/R2. If you call it a ratio and then you put labels as those in Fig.1 1K-M, the reader can easily be misled. At first, I was very confused by looking at those panels, I did not understand why the ratio was always < 1, even if the evoked activity should have been larger than the spontaneous activity. Then I remembered that with “ratio” the authors did not mean a real ratio, but the index defined here in the methods. But this is terribly confusing. This is not a ratio, it is an index. The authors should call it an “evoked rate index” or whatever they like that, but they should not call it a ratio. More importantly, they should write in the labels of Figs. K-M this name they will come about and not stuff like “Ev/Sp Awake” because otherwise the reader will think that axis reports what they wrote on it: the ratio Ev/Sp, which, unfortunately, it is not what the axis shows.

8. Line 225. It is not said explicitly, but it looks like the authors take the time of the peak of the response (relative to stimulus onset I guess - again, this is not specified explicitly) as the response latency. This is unusual, since the neurons obviously start to fire before the FR reaches its peak. This is why, most studies take as the response latency the time at which the driven (i.e., background subtracted) FR increases of a given percentage or of a given number of SD, relative to the background. See, for instance, these articles (Brincat and Connor, 2006; Zoccolan et al., 2007) for monkey studies and this article for a rat V1 study (Vermaercke et al., 2014). I would advise the authors to do the same, otherwise comparisons with latencies reported in other studies (e.g., (Vermaercke et al., 2014)) would be difficult.

9. Lines 283-288. Here is one of the many parts where the writing and the logic of the description must be improved. The authors first refer to panel D, where data from a single session with 17 simultaneously recoded cells are shown, then they comment panel C (where the full data set is shown, as far as I can tell) and then in their final sentence they comment again on the variety of behaviors cells may display within a single ensemble. This is confusing. They should follow the logic of their figure: comment first panel C (full data set), then panel D (single ensemble of 17 cells).

10. Lines 308: “all recorded units revealed a bimodal distribution (Figure 1F)”. It looks that the assessment of bimodality was done only qualitatively, by visual inspection. It would be better if the authors used a more quantitative and object approach. I suggest either running a clustering algorithm (e.g., k-means with k = 2) or trying to fit the density of cells on the plane defined by the axes of Fig. 1F with two 2-d Gaussians, and then assign the cells to either Gaussian based on their proximity to their center (in terms of units of SD).

11. Line 321: “The GABAergic antagonistic anesthestic regimes (Isoflurane and Isoflurane/Dormicum)”. This should be an agonistic (not antagonistic) regime, as far as I underdtand.

12. Lines 322-323. Here the authors for the first time segregate their results based on the kind of anesthetics they uses, and they report radical differences. But what about the previous figures (panels A-G)? It is not specified whether the reduction of FR reported in those panels was obtained with isoflurane or ketamine. We should assume that those data were obtained with isoflurane? But this should be specified clearly in describing panels A-G. Similarly, do all the panels in figure 1 up to panel J refer to evoked or spontaneous FR? This is not told. At some point a full section starts (line 333) about the difference between spontaneous and evoked activity, but what about the data shown before? To what part of the response do they refer to?

13. Line 337: the authors should avoid jumping ahead of their figures, making a quick reference to Fig. 2F (without explaining what it shows) while describing Fig. 1 still. This is extremely confusing.

14. Lines 344-346: “our results support the proposed effect from previous investigations that an effect of anesthesia on unit activity is mainly a reduction in spontaneous activity”. This is all very confusing. The explanation of these panels in Fig.1 is hard to follow because too many bits of information are missing or are scattered through too many paragraphs. The issues are:

- In panels A-G we are told that anesthesia produce a reduction of FR

- In panel H we leaned that not all anesthetics produce such a reduction: only those based on isoflurane? But then, I wander: was the reduction of FR shown in panels A-G present because those data refer to isoflurane anesthesia? Impossible to know because this information is missing.

- Finally, in panels K-M, we learn that there is a differential effect of the anesthetics on spontaneous vs. evoked activity. But then I wonder: were the data shown in panels A-J referring to spontaneous or evoked FR. Impossible to know because this information is missing.

To complicate even more things, in panel M we see that also for ketamine there is a significant effect of anesthesia: larger ev/sp ratio than awake. But didn't ketamine failed to produce any reduction of FR (panel H)?

Finally, in the sentence above, references are missing.

15. Line 366: “after trough (awake: 168 {plus minus} 49 ms, anesthesia: 307 {plus minus} 140 ms, p=0.003, Wilcoxon).” make reference to Fig. 2B here!

16. A very important issue of this study is that large parts of the figures are orphan of any comments or any description in the main text. An example is Fig. 2E and Fig. 2F (barely mentioned). But here, it would be important to understand what the figures mean and imply. For instance, in the case of Fig. 2F, there are many issues:

- the y label says: “# units”, but then the scale goes up to 50,000. So from the legend I understood that each line is actually a trial of a unit and all trials of all units are shown. Is it correct? If so, this should be more explicitly said in the text.

- there seem to be some raking of the units, but according to what exactly? Overall FR? Spontaneous FR? Evoked FR? This should be written somewhere.

- Finally, and more importantly. We are told in Fig. 1 that ketamine does not significantly reduce FR. But here it looks like it does dramatically reduce FR. more than any other anesthetic, in both the spontaneous and evoked phase of the response. How is it possible this incongruence? Is it possible, by any chance, that the authors have managed to mislabel the figure and the green dots actually refer to isoflurane, while the blue dots to ketamine?

17. Lines 378-380. It looks like the authors are making reference to Fig. 4B, while they should have made reference to Fig. 2F and G, and they made reference to Fig. 4C, while they should have made reference to Fig. 2H. Well, this is my guess at least ...

18. Lines 379-380: “normalizing the firing rates to baseline levels”. How was this normalization done exactly? Why are curves in H starting from negative values?

19. Line 391: “latency under anesthesia was largely independent of latency in the awake state.” It is ok to report this result for the latency of the peak of the response, but this analysis should be done also on the actual latency of the response (see my comment #4).

20. Section starting at Line 407. This comments applies here and to the many other places of the manuscript where the authors do not comment some of the panels of their figures. If you have decided to show Fig 3A, you must describe it in the text. You cannot simply jump to Fig. 3B. If you do not feel the need to describe Fig. 3A, then why have you shown this panel? Do not even bother showing it then. Or describe it and explain it to the reader.

21. Outliers were removed from figure 3C, as mentioned at line 412. Which was the method for outliers identifications? Usually should be based on distribution sigma (for example all data exciding 10 SD are classified as outliers)

22. Line 427. If Fig 3F is the next figure you want to describe then call it Fig. 3D. But do not jump to describe panel F without having described panels D and E. This is very bad writing.

23. Line 450: “We find a higher CV in the awake state (Figure 3G”. Here as in many other figures where the anesthetic is not specified: do these data refer to neurons recorded under all three anesthesia conditions? Or just one specific anesthetic? The labels of the figures, the legends and the main text should all be revised so that the reader can immediately and effortlessly know under what kind of anesthesia the data were recorded.

24. Section titled “Preservation of temporal sequences”. The result about the preservation of the temporal sequence of neuronal firing in awake and anesthetized state and among the two is presented as one of the main findings of the paper. I agree that this can potentially be an interesting result, but, although I was able to follow the overall meaning of this section and of figure 4, many of the details of the analyses are unclear. So this section needs to be fully revised to make it intelligible, by describing step by step (in sequential order) the panels of Fig. 4, and explaining carefully why the analysis shown in a panel differ from the analysis shown in the previous and next one. Also, it is not enough to make reference to the literature when explaining your method/metric - you must fully describe it.

For instance, from the description of Fig. 4C provided in the main text, it seem that the bar plots refer to CC between the latencies of the units within an ensemble in two different states/sessions. But how this is different from what the authors later call MSL? And why, in the legend of Fig. 4C, these CC are called MSL? And are these MSL in Fig. 4C the same of those shown in Fig. 4B? And why, again and again, is Fig. 4C described before Fig. 4B? Do we need Fig 4B to understand Fig 4C? Is there any reason why one panel comes before the other?

To summarize: this is, as usual, very confusing. I do not have the patience and the strength of trying to figure out something so obscure. I can only suggest to the reviewers two things:

- provide a detailed explanation of the metrics you compute (here as everywhere else), possibly with the aid of some apposite figures.

- find one or more colleagues (better more) that befriend you and ask them to read through your manuscript and honestly tell you if they understand what you are trying to explain. Engage in long discussions with them until you manage to put together some intelligible text.

25. Lines 511-513. “More populations are included in this measure versus the single population- MSL rank correlations because the high demand of unit number for MSL individual spike times in sequence relevant correlation comparisons.” This is simply one of the most unclear sentences I ever happed to read!

26. Lines 521-522. “As expected, for all measures the shuffling resulted in no skewness and normal distribution of events”. Is that really true? At least for the first and third panel (especially for the third), the yellow bar plots seem positively skewed. The authors should quantify the skewness of the yellow bar plots, compare it to the skewness of the gray bar plots and compare the two distributions statistically. My impression is that a fraction of the sequence effect is preserved after shuffling.

27. Lines 548-549. “each grating cycle size was estimated at the location furthest and closest from the screen to the rat's eyes”. Why the need of estimating a max and min SF. If the authors tracked the rat head I assume that they did not just track the position but also the direction where the head was pointing. This should allow estimating precisely the actual SF of the stimuli. To do this is necessary just to track two points (LEDs). Did the author really only track one single LED, knowing the kind of analysis they planned to do?

28. Lines 561-564. I can understand that the fig. 5D shows a preservation of activity for neurons preferring lower SF, but not high TF, but I do not understand how this figure relates to the claim of this sentence.

29. Lines 572-573: “unit preferences was found between awake and anesthesia (Figure 5H,I)”. Again, if panels H and I are what you want to describe next, why don't you just name them panels E and F?

30. Section titled “Modelling awake-anesthesia data”. I don't feel that the modeling is adding anything to this study. It is a too simplistic model of visual cortex to be meaningful. A model is useful if it shows an emerging property. Is this the case? Is it any unexpected that by increasing the inhibition in the network the spontaneous and evoked responses are lower? I don't think so. What do we learn about the processing in the network? My opinion is that the whole modeling work should be dropped, unless the authors can convincingly show that we can learn something from it.

31. There are a few things reported in the results that are not properly discussed.

- The latencies in the results (Lines 365, 383-387) are discussed in the paragraph starting at line 701, but focusing only on the relative increase/decrease across brain states. No comparison of absolute values during anesthesia and wakefulness is made with values in other studies. Are the obtained values comparable with what reported for visual neurons in literature?

- No comparison with literature is reported for the delays measure in the ketamine/xylazine condition (L 725)

- Always on latencies, the studies cited in the paragraph starting at line 727 are from different species (Angel et al 1973 on Rabbits, Ter-Mikaelian et al 2007 on Mongolian gerbil, Cheung et al 2001 on Cats). First of all it should be specified, but things should be compared with studies on visual (and other sensory stimuli) in anesthetized rats, as there are studies that report latencies on rats' V1.

- In line 283-284, the authors state that “neighboring neurons may respond differently to the same level of anesthesia.” This affirmation should be properly discussed and framed in the wide range of literature on the topic.

- Same thing for the statement at lines 369: “the LFP signature is not only shifted in time, but appears to last longer during anesthesia ”; and at line 430: “activity more correlated when there is no stimulus”. Those are all arguments extensively studied and should be discussed a bit more extensively.

- A suggestion for the authors of a paper on neural correlation and synchrony across areas under different levels of anesthesia in rats: Bettinardi et al, Neuroimage 2015

32. General comment for the Discussion: make again reference to specific figure panels while discussing specific results.

33. Lines 713-714. “on separate pools of an unknown number of units.” What does it man “unknown?” in this paper, is the number of units really not reported? Please double check.

34. Minor problems in the figures:

1A - color legend missing

2E - axis labels missing

3A - colorbar label missing

3D, left panel - time and amplitude units missing

4B - colorbar label missing

4C - x axis label and color-code label missing

4E - x axis label missing

Reviewer 2:

1. Why did the experimenters chose the three types of anesthesia? It appears for a systematic approach relating to a host of previously generated data under anesthesia, at least the fentanyl condition and also urethane should be added.

2. The stimulus control is worrisome. The authors compare conditions in which an anesthetized animal was simply placed in front of a screen with situations, in which a mobile awake animal was roaming in front of the screens. They chose to pick the intervals in which the animal was sessile. However, only head position was assessed for this purpose. What about other behaviorally important parameters. What were the animals doing while being sessile? Sleeping, drowsing, grooming, etc.? In none of the reported situations eye / gaze position was monitored. Nevertheless, the authors present firing rates at a high temporal resolution (milliseconds). What is the effect of different spatial frequencies of the stimulus and receptive field position on the grating on latency and pattern estimations like the one presented in Figs. 2 and 4? Why wasn't at least the distance varied in anesthetized experiments.

3. Figure 1: What is meant with anesthesia? An average of all three types? I seriously doubt that this makes sense as in the rest of the paper several significant differences between these regimes are shown. I strongly recommend to abolish all averages across anesthesia regimes from the manuscript!

4. Figure 4 and related text: This whole section needs to be reworked completely. It is barely intelligible. The analysis related to patterning has to be succinctly explained here. What is 'average mean (!) spike latency' ???

5. In this section the impact of lack of stimulus control is most critical. The authors need to be very meticulous here and argue well which of their results and interpretations still stand when they consider (perhaps systematic) different stimulus conditions in the awake vs. the anesthetized case (eye / whole body movements, distance / angle to the screen etc.).

Minor comments

1. What is a 16 electrode tetrode array? 4 tetrodes?

2. Figure 1: What is the n e.g. in panel B and G? Trials? Spikes? Why is the n only given for one group in panel B?

3. Fig. 1D: Abscissa label?

4. Fig. 1G: Show median and iqr (or similar) as well!

5. Spike types: Demonstrate example waveforms form all three types (BS, NS, triphasic).

6. Line 346: The statement warrants references!

7. Figure 3D: The abbrev. BS was broad spiking before. What is the purpose of this panel anyway? In a burst suppression situation, enhanced correlation is highly expected!

8. Fig. 4 is inaccessible! Referencing other papers is not good enough here. Explain all strategies of analysis here in the text!

9. Fig. 5D: Axes labels?

Additional minor changes that I found:

Lines 355-357: completely unclear. Please revise. Maybe also be OK to completely remove and just proceed to the section on “Visually evoked latencies are delayed under anesthesia”

Lines 347-353 seem inconsistent with line 289-297 that state that only sessile sessions were analyzed.

Line 280: the firing rates were ... (instead of was)

Line 292-293: text is not clear, and seem to disagree with Neil and Stryker results.

Line 329: consider the wording such as “had most stable response dynamics” instead of “has the lowest changes in firing rate”

Line 381 ... stimulus onset was ...

Line 459: “compared to awake state.”

## Author Response

Synthesis Statement for Author (Required):

The study reports a set of very systematic and comprehensive measurements of the impact of different kinds of anesthetics on the activity of different cell types in the brain. The results will have important impact on the field. However, both reviewers felt strongly, and I agree, that the presentation needed substantial improvements including figure re-arrangements. The sections that were especially not clear are on rank correlations. The detailed comments are below. Also, some discussion is needed as to the choice of particular anesthetics used, and some additional detail to characterize the “sessile” state. Further, the work by Churchland et al. Nature Neuroscience 2010 is relevant to results described on lines 425-432.

We have made adjustments to the text and figures in response to the majority of the reviewers concerns.

Reviewer 1:

In this study, the authors compare some neuronal response properties of rat V1 neurons in the anesthetized and awake state. These properties include magnitude of the spontaneous and evoked responses, latency of the response and the temporal pattern of firing of ensembles of simultaneously recorded neurons. The main strength of the study is the fact that it based on chronic recordings from implanted animals, which were tested in either the awake or anesthetized state, thus allowing probing the same neurons under both states. In addition three different kinds of anesthetics were used. On the other hand, the main weakness of the study is that the writing is quite poor, especially in the Results section, where not only analyses and results are described approximately, but the material presented in the figures is not described in sequential order: the authors jump back and forth among different panels of a figure or even among different figures, sometime mislabeling the figures they refer to in the main text. This has made the Results extremely difficult and tiring to read, with the consequence of lowering considerably the understanding of the authors' conclusions. As a consequence, I found myself unable to fully gauge the solidity and originality of the findings reported by the authors. On top of that, I also found that the discussion of similar results form previous studies is too slim and does not quite cover the existing literature.

In any event, given that the experiments the authors carried out seem to be solid enough (although not perfect in their design: see the lack of tracking of head direction in the awake recordings), and given the fact that the topic is of some interest (not many papers have followed the same neurons across awake and anesthetized states), I think that the manuscript deserve the chance to be revised and reconsidered. But this revision needs be very deep and careful: 1) the order of the figures should be totally revised, making it sequential and aligned to the description provided in the text; 2) the metrics used in the analysis should be completely described in the Methods (it is not enough to refer to previous studies that have used them) and also described again (shortly) in the Results, in the first instance that they are used; 3) and the whole text should be revised by some colleague of the authors, possibly mother-tongue, that should critically read the full manuscript and honestly point to the authors what she/he does and does not understand. Only after that this is done, and the manuscript has been considerably improved, it will be possible to truly assess the solidity and impact of the manuscript.

Below are my specific comments.

1. Introduction and Discussion: the recent article of Durand et al (J Nerosci, 2016) should be mentioned somewhere in the introduction and then discussed at length in the Discussion, since that articles address many of the same questions investigated by the authors. The authors should compare the two studies and explain what similarities and differences are found across them.

Thank you for pointing our attention to Durand et al (2016) which is very relevant to discuss in this paper. The paper came out during the submission phase of the current manuscript and was regrettably not included for this reason. We have now included it in the introduction and discussion.

2. Lines 210-211: “Evoked firing rates were calculated as the maximum firing rate of each unit across all bins following stimulus presentation”. What bins are the authors referring to here? I guess they binned the time axis and computed the average firing rate (FR) in consecutive time bins, but it is not mentioned the width of these bins; neither that the time axis has been binned. The authors cannot simply assume that the reader will know what they are talking about. They need to provide all the details of their methodological approach, and possibly in a sequential, logical order.

We apologize for our lack of clarity on this point. This refers to the binning of the time axis of the PTSH. Adjusted in the manuscript on line 219-223.

3. It was not clear at which cortical depth the presented data were collected. The authors state in the methods that tetrodes were implanted “into the primary visual cortex, about 300 um below the dura mater at an angle of 30-40 degrees in the lateral to medial direction” (L102). Then they point out that the electrodes could be easily moved up and down and that “the recording location was extrapolated from deepest trace identified by histological inspection of the sections and the tetrode-tuning log” (L 157-168). Thus is not clear whether the recorded neurons are all from the same layer, or are presented independently from their depth. From what I understood, most likely recordings come from layer 2/3. This issue should be explained by the authors as, especially in the anesthesia state, this could affect the results. More importantly, if the authors sampled multiple layers, they should try to segregate their data also on a layer-by-layer fashion. If they cannot do so, they should explain why.

Since the tetrodes can be moved quite far we did indeed sample across all layers. We initially wanted to include layer-specific results, but the magnitude of the work was in the end considered too large to effectively communicate our main aim. We might want to include these results in a separate paper.

However, we are glad the reviewer raises this point since we would like to specify how our data is distributed across the layers. We have now added this piece of information. See line 298-300 in the results.

4. In the methods, the author state that the Isoflurane concentration was set at 1.5% for the condition 'Isoflurane only“ because this was the ”minimum alveolar concentration for adult rats (Mazze et al. 1985), while for the Isoflurane/Dormicum “conditions were initially tested with pilot studies to achieve the lightest possible level of anesthesia” (starting from line 120, to line 130). As also the authors seem to be aware of (because they adjust the concentrations), the correspondence between depth of anesthesia and % of isoflurane varies a lot across animals, and across studies in the literature. For example, 1.5% is reported as “light anesthesia” in Hudetz et al 2003, while is defined as “deep” in Silva et al 2010 (Silva and colleagues define light anesthesia at 0.8% Isoflurane). I would specify this in the methods and report a wider range of literature, with some more recent study.

As noted by the reviewer, objective measurements of the level of anesthesia is not trivial. The paper we cite by Mazze et al (1985) systematically tested how isoflurane levels corresponds with anesthetic depth measured through continuous reflex testing. The paper suggested by the reviewer (da Silva et al 2010) did not test reflexes but measured other parameters such as blood iso- concentration and heart rate. As such the level of anesthesia might be lower than surgical levels of anesthesia with this paper. Considering whether the aim is to achieve surgical levels of anesthesia, or purely immobilization, the level of isoflurane anesthesia is a subject of variation, as the reviewer is aware. It is thus difficult to compare anesthesia levels across different papers using different end-criteria (blood content vs intact reflexes). In our study however, the aim was to study the effect of the standard anesthesia levels used commonly in visual labs on visual processing compared to awake animals. The anesthesia level was adjusted for each individual animal as is standard protocol for the body of literature for which we aim for our results to be relevant. Since we use a custom-made made mask as well as a standard gas-retractor (pulling the gas and exhalation-waste away through a separate tube), we expect that the actual concentration of isoflurane is lower for the animal. Silva et al (2010) used chamber level monitoring with exact % values and thus has more control over the anesthetic-level. Also, the Hudetz et al (2003) paper uses additions of O2 and NO2 to optimize the stability of the anesthesia. Based on our pilot experiments, we know that we could not achieve a stable anesthesia with lower % for the first 30 min (see line 123 in the original manuscript). As we state, the isoflurane condition was also adjusted to lower % levels throughout the experimental session when the rat displayed signs of deeper anesthesia (heavier breathing, lower heartrate, and burst-suppression EEG). For this reason, we expect the isoflurane % levels to be around 1-1.5 % for the first 15-20 minutes, since this was the minimum level we could achieve with the animal and lowered to 0.8-1% with increasing time and indications of deeper anesthesia.

5. The author reported the “low signal-to noise ratio” as a method for units exclusion from the analysis (L 208). This should be specified better. What was the threshold for exclusion? The SNR was calculated on spontaneous or stimuli-evoked activity?

Corrected so that all units needed to pass an SNR of 2.75 (Smith & Kohn, 2008) to be included in the analysis. See line 217 in the method section. In addition, since spike waveforms do not differ between spontaneous and evoked time-periods, the analysis was conducted on overall firing rate.

6. The units selection reported at line 229-232 is the same of point 4? Please clarify .

We have added a more specific description of the purpose of this exclusion criteria. See line 241-243.

7. Lines 214-215: “The ratio between maximum evoked rate (R1) and spontaneous rate (R2) was calculated as calculated as (R1-R2)/(R1+R2) for each unit”. This should not be called a ratio. It is confusing. A ratio would be R1/R2. If you call it a ratio and then you put labels as those in Fig.1 1K-M, the reader can easily be misled. At first, I was very confused by looking at those panels, I did not understand why the ratio was always < 1, even if the evoked activity should have been larger than the spontaneous activity. Then I remembered that with “ratio” the authors did not mean a real ratio, but the index defined here in the methods. But this is terribly confusing. This is not a ratio, it is an index. The authors should call it an “evoked rate index” or whatever they like that, but they should not call it a ratio. More importantly, they should write in the labels of Figs. K-M this name they will come about and not stuff like “Ev/Sp Awake” because otherwise the reader will think that axis reports what they wrote on it: the ratio Ev/Sp, which, unfortunately, it is not what the axis shows.

We agree that the use of the term “ratio” was misleading. Ratio has now been changed to index in text and figures. See line 226-231 in methods, and 354-370 in results for rewritten paragraph.

8. Line 225. It is not said explicitly, but it looks like the authors take the time of the peak of the response (relative to stimulus onset I guess - again, this is not specified explicitly) as the response latency. This is unusual, since the neurons obviously start to fire before the FR reaches its peak. This is why, most studies take as the response latency the time at which the driven (i.e., background subtracted) FR increases of a given percentage or of a given number of SD, relative to the background. See, for instance, these articles (Brincat and Connor, 2006; Zoccolan et al., 2007) for monkey studies and this article for a rat V1 study (Vermaercke et al., 2014). I would advise the authors to do the same, otherwise comparisons with latencies reported in other studies (e.g., (Vermaercke et al., 2014)) would be difficult.

We used the time from stimulus onset to the maximum firing rate as measure of latency (similar to Pisauro et al 2013, and Brincat & Connor 2006). We appreciate the reviewers suggestion of calculating the latency to stimulus onset, as this would serve to validate our findings even better. We have added the stimulus onset analysis from Brincat & Connor (2006) to our figure 2J & K, added a description in the method section at line 245-247. Further, we have described the findings in the results section in line 409-416 and discussion line 771-773.The method for onset latency from Vermaecke et al. (2014) was tried, but due to very high criteria for the stimulus-evoked activity (3 SD from baseline), many units did not reach criteria. Of this reason, we chose to use the Brincat & Connor version, and direct comparison of latency numbers is thus difficult with the Vermaecke rat study. More importantly however, we feel the aim of our study is to show how each unit display variations in latency between states while keeping the analysis and sampling identical. Since analysis methods on latency often relies on different baseline criteria like the reviewer mentions, this undoubtedly creates variations between studies and difficulty in comparing the numbers directly.

9. Lines 283-288. Here is one of the many parts where the writing and the logic of the description must be improved. The authors first refer to panel D, where data from a single session with 17 simultaneously recoded cells are shown, then they comment panel C (where the full data set is shown, as far as I can tell) and then in their final sentence they comment again on the variety of behaviors cells may display within a single ensemble. This is confusing. They should follow the logic of their figure: comment first panel C (full data set), then panel D (single ensemble of 17 cells).

We agree that this structure is not the most straight forward way to present the results. We have adjusted the text to fit with the reviewer's suggestion.

10. Lines 308: “all recorded units revealed a bimodal distribution (Figure 1F)”. It looks that the assessment of bimodality was done only qualitatively, by visual inspection. It would be better if the authors used a more quantitative and object approach. I suggest either running a clustering algorithm (e.g., k-means with k = 2) or trying to fit the density of cells on the plane defined by the axes of Fig. 1F with two 2-d Gaussians, and then assign the cells to either Gaussian based on their proximity to their center (in terms of units of SD).

We are glad to get the chance to correct this. K-means and linkage clustering divided the units inappropriately, so we assigned it to a threshold of peak-amplitude width to cluster the units. However, since our wish is for our data to be as clear as possible, we have adjusted the measure to the two 2D gaussian fit that the reviewer suggests. This solution appropriately assigned the narrow spiking and broad spiking units and we are very thankful for the suggestion. See line 214-215 of the method section and adjusted numbers on lines 325-331 of the results.

11. Line 321: “The GABAergic antagonistic anesthestic regimes (Isoflurane and Isoflurane/Dormicum)”. This should be an agonistic (not antagonistic) regime, as far as I underdtand.

Corrected.

12. Lines 322-323. Here the authors for the first time segregate their results based on the kind of anesthetics they uses, and they report radical differences. But what about the previous figures (panels A-G)? It is not specified whether the reduction of FR reported in those panels was obtained with isoflurane or ketamine. We should assume that those data were obtained with isoflurane? But this should be specified clearly in describing panels A-G. Similarly, do all the panels in figure 1 up to panel J refer to evoked or spontaneous FR? This is not told. At some point a full section starts (line 333) about the difference between spontaneous and evoked activity, but what about the data shown before? To what part of the response do they refer to?

We have tried to make this clearer in text and figures. Although we recognize the lack of specificity of the general anesthetic grouping, we feel that it is appropriate to compare the effect of anesthesia as a whole before going into specifics about the different anesthetics. Since the specific mechanisms of anesthesia are not fully known, it is also unknown whether the various anesthetics that have different modes of action, share traits on the effect of single units. Therefore, we see it as relevant to highlight their action collectively, before going into how they differ. However, we agree that this should be specified more clearly in the text and legends, and will change accordingly.

The reference to firing rates as a general measure without the initial classification into spontaneous and evoked responses is very common, however we have added a line to the method section. See line 208-209, 287-289, 333-335 for changes.

13. Line 337: the authors should avoid jumping ahead of their figures, making a quick reference to Fig. 2F (without explaining what it shows) while describing Fig. 1 still. This is extremely confusing.

We agree and the reference to Figure 2 F is moved to its correct place.

14. Lines 344-346: “our results support the proposed effect from previous investigations that an effect of anesthesia on unit activity is mainly a reduction in spontaneous activity”. This is all very confusing. The explanation of these panels in Fig.1 is hard to follow because too many bits of information are missing or are scattered through too many paragraphs. The issues are:

- In panels A-G we are told that anesthesia produce a reduction of FR

- In panel H we learned that not all anesthetics produce such a reduction: only those based on isoflurane? But then, I wander: was the reduction of FR shown in panels A-G present because those data refer to isoflurane anesthesia? Impossible to know because this information is missing.

Corrected (see point 12 for description of changes).

- Finally, in panels K-M, we learn that there is a differential effect of the anesthetics on spontaneous vs. evoked activity. But then I wonder: were the data shown in panels A-J referring to spontaneous or evoked FR. Impossible to know because this information is missing.

See point 12 for our response and description of changes.

To complicate even more things, in panel M we see that also for ketamine there is a significant effect of anesthesia: larger ev/sp ratio than awake. But didn't ketamine failed to produce any reduction of FR (panel H)? -

We are sorry for not explaining our findings in a more understandable manner and we are grateful for getting the chance to specify it more clearly. Although ketamine did not induce an overall change in firing rate, there was a significant change in the evoked-spontaneous index. I.e. since the ket/xyl index-change occurs despite relatively unchanged firing rates, the overall tendency is that evoked activity for the majority of units increase while spontaneous activity decrease. In contrast, for the iso-based anesthetics, both evoked and spontaneous activity decrease. However, spontaneous activity decreases more than evoked, thus creating a similar change in the index This suggest that the index is more reliable to detect the anesthesia-mediated changes on responses to evoked and spontaneous activity specifically. This is now stated in line 363-365 and we have rearranged the paragraph to make it easier to read.

Finally, in the sentence above, references are missing.

Corrected.

15. Line 366: “after trough (awake: 168 {plus minus} 49 ms, anesthesia: 307 {plus minus} 140 ms, p=0.003, Wilcoxon).” make reference to Fig. 2B here!

Corrected.

16. A very important issue of this study is that large parts of the figures are orphan of any comments or any description in the main text. An example is Fig. 2E and Fig. 2F (barely mentioned). But here, it would be important to understand what the figures mean and imply. For instance, in the case of Fig. 2F, there are many issues:

In the revised manuscript we have made sure that all figures are mentioned in the main text.

- the y label says: “# units”, but then the scale goes up to 50,000. So from the legend I understood that each line is actually a trial of a unit and all trials of all units are shown. Is it correct? If so, this should be more explicitly said in the text.

Label on the y axis is now corrected to # Trials and described in the result section.

- there seem to be some ranking of the units, but according to what exactly? Overall FR? Spontaneous FR? Evoked FR? This should be written somewhere.

We have added the following description to the figure legend and results: “Units are ranked according to awake firing rates from high to low rate (bottom up)”.

- Finally, and more importantly. We are told in Fig. 1 that ketamine does not significantly reduce FR. But here it looks like it does dramatically reduce FR. more than any other anesthetic, in both the spontaneous and evoked phase of the response. How is it possible this incongruence? Is it possible, by any chance, that the authors have managed to mislabel the figure and the green dots actually refer to isoflurane, while the blue dots to ketamine?

Corrected.

17. Lines 378-380. It looks like the authors are making reference to Fig. 4B, while they should have made reference to Fig. 2F and G, and they made reference to Fig. 4C, while they should have made reference to Fig. 2H. Well, this is my guess at least.

Figure reference corrected.

18. Lines 379-380: “normalizing the firing rates to baseline levels”. How was this normalization done exactly? Why are curves in H starting from negative values?

We described this normalization in the methods section: “Additionally, a normalized firing rate was calculated for each unit, where firing rates of each bin was normalized to the mean firing rate (R2) of the unit (R1) (R1-R2)/(R1+R2)”. We have also added this description to the results. See line 402-403.

19. Line 391: “latency under anesthesia was largely independent of latency in the awake state.” It is ok to report this result for the latency of the peak of the response, but this analysis should be done also on the actual latency of the response (see my comment #4).

We have added a stimulus-onset latency measure. See response to point 8.

20. Section starting at Line 407. This comments applies here and to the many other places of the manuscript where the authors do not comment some of the panels of their figures. If you have decided to show Fig 3A, you must describe it in the text. You cannot simply jump to Fig. 3B. If you do not feel the need to describe Fig. 3A, then why have you shown this panel? Do not even bother showing it then. Or describe it and explain it to the reader.

Corrected.

21. Outliers were removed from figure 3C, as mentioned at line 412. Which was the method for outliers identifications? Usually should be based on distribution sigma (for example all data exciding 10 SD are classified as outliers)

We appreciate this suggestion. The outliers are now removed from the graphical presentation if they exceed 5 standard deviations from the mean.

22. Line 427. If Fig 3F is the next figure you want to describe then call it Fig. 3D. But do not jump to describe panel F without having described panels D and E. This is very bad writing.

Thank you for this correction. We have adjusted the figure order to match the buildup of the main text.

23. Line 450: “We find a higher CV in the awake state (Figure 3G”. Here as in many other figures where the anesthetic is not specified: do these data refer to neurons recorded under all three anesthesia conditions? Or just one specific anesthetic? The labels of the figures, the legends and the main text should all be revised so that the reader can immediately and effortlessly know under what kind of anesthesia the data were recorded.

As described under point 12, we feel it is within our aims to generalize about effects of anesthesia as a whole before going into how the various types of anesthesia impacts the effect. The next figure (Figure 3H) describes the CV for the different anesthetics. We have included a line specifying that for figure 3G the CV is calculated on all anesthetics. See line 479.

24. Section titled “Preservation of temporal sequences”. The result about the preservation of the temporal sequence of neuronal firing in awake and anesthetized state and among the two is presented as one of the main findings of the paper. I agree that this can potentially be an interesting result, but, although I was able to follow the overall meaning of this section and of figure 4, many of the details of the analyses are unclear. So this section needs to be fully revised to make it intelligible, by describing step by step (in sequential order) the panels of Fig. 4, and explaining carefully why the analysis shown in a panel differ from the analysis shown in the previous and next one. Also, it is not enough to make reference to the literature when explaining your method/metric - you must fully describe it.

We have now included a better step-by-step description of the procedures.

For instance, from the description of Fig. 4C provided in the main text, it seem that the bar plots refer to CC between the latencies of the units within an ensemble in two different states/sessions. But how this is different from what the authors later call MSL?

And why, in the legend of Fig. 4C, these CC are called MSL? And are these MSL in Fig. 4C the same of those shown in Fig. 4B? And why, again and again, is Fig. 4C described before Fig. 4B? Do we need Fig 4B to understand Fig 4C? Is there any reason why one panel comes before the other?

To summarize: this is, as usual, very confusing. I do not have the patience and the strength of trying to figure out something so obscure. I can only suggest to the reviewers two things:

The text is now improved to avoid this confusion.

- provide a detailed explanation of the metrics you compute (here as everywhere else), possibly with the aid of some apposite figures.

Apposite figures have been included.

- find one or more colleagues (better more) that befriend you and ask them to read through your manuscript and honestly tell you if they understand what you are trying to explain. Engage in long discussions with them until you manage to put together some intelligible text.

The manuscript has been revised in order improve the text.

25. Lines 511-513. “More populations are included in this measure versus the single population- MSL rank correlations because the high demand of unit number for MSL individual spike times in sequence relevant correlation comparisons.” This is simply one of the most unclear sentences I ever happed to read!

We agree, and the sentence has been rephrased.

26. Lines 521-522. “As expected, for all measures the shuffling resulted in no skewness and normal distribution of events”. Is that really true? At least for the first and third panel (especially for the third), the yellow bar plots seem positively skewed. The authors should quantify the skewness of the yellow bar plots, compare it to the skewness of the gray bar plots and compare the two distributions statistically. My impression is that a fraction of the sequence effect is preserved after shuffling.

We appreciate the suggestion of statistically confirming the lack of skewness for the shuffle data. The shuffling of unit identity did indeed remove skewness for all measures. We have added significance testing to a theoretical mean of zero to verify our findings. The real data produces p-values of p<0.001 while the shuffle data yields non-significant p-values. In addition, a non-significant ANOVA between the groups on the shuffle data is reported.

27. Lines 548-549. “each grating cycle size was estimated at the location furthest and closest from the screen to the rat's eyes”. Why the need of estimating a max and min SF. If the authors tracked the rat head I assume that they did not just track the position but also the direction where the head was pointing. This should allow estimating precisely the actual SF of the stimuli. To do this is necessary just to track two points (LEDs). Did the author really only track one single LED, knowing the kind of analysis they planned to do?

Ideally we would have preferred to track at least two LED points on the rats head, and we did indeed try. However, in our experimental setup with the glass box and visual stimulation we got too much reflections and too poor signal-to-noise ratio with two differently sized LEDs. Thus, we wanted to highlight how the maximum and minimum distance would impact the results, and the different positions of the animal could not account for a big enough change in the perceived spatial frequency to alter any conclusions.

28. Lines 561-564. I can understand that the fig. 5D shows a preservation of activity for neurons preferring lower SF, but not high TF, but I do not understand how this figure relates to the claim of this sentence.

The sentence has been rephrased.

29. Lines 572-573: “unit preferences was found between awake and anesthesia (Figure 5H,I)”. Again, if panels H and I are what you want to describe next, why don't you just name them panels E and F?

We have restructured the text to accommodate the figure layout.

30. Section titled “Modelling awake-anesthesia data”. I don't feel that the modeling is adding anything to this study. It is a too simplistic model of visual cortex to be meaningful. A model is useful if it shows an emerging property. Is this the case? Is it any unexpected that by increasing the inhibition in the network the spontaneous and evoked responses are lower? I don't think so. What do we learn about the processing in the network? My opinion is that the whole modeling work should be dropped, unless the authors can convincingly show that we can learn something from it.

We find the modelling part of the paper to be important as it links our findings to cortical network models developed by the computational neuroscience community. The model of choice, the Brunel network with recurrently connected populations of excitatory and inhibitory neurons, has substantial merit as it successfully describes the observed “asynchronous, irregular state” of cortical networks. We thus find the question of to what extent the observed effects of anesthesia can be understood by assuming its main effect to be increased inhibition in a Brunel network, to be both relevant and interesting. As it turns out some the observed effects are observed qualitatively in the network simulations (reduced firing rates, changes in pairwise correlation and coefficient of variation), others are not (shift in latency). This approach follows in the tradition of physics and engineering where hypotheses are formulated precisely in terms of mathematics and predictions from the mathematical model is compared with experimental data. The modeling results also provide predictions for future experiments about how the distribution of membrane potentials will be affected by anesthesia in the neuron populations.

31. There are a few things reported in the results that are not properly discussed.

- The latencies in the results (Lines 365, 383-387) are discussed in the paragraph starting at line 701, but focusing only on the relative increase/decrease across brain states. No comparison of absolute values during anesthesia and wakefulness is made with values in other studies. Are the obtained values comparable with what reported for visual neurons in literature?

The latency parameters correspond well to previous literature using the same peak-latency measure (e.g. Pisauro et al 2013, and Brincat & Connor 2006). We have added a description of absolute values in line 765-771. However, see point 8 for our response to the difficulty in comparisons between studies.

- No comparison with literature is reported for the delays measure in the ketamine/xylazine condition (L 725) .

Please see our response under point 8.

- Always on latencies, the studies cited in the paragraph starting at line 727 are from different species (Angel et al 1973 on Rabbits, Ter-Mikaelian et al 2007 on Mongolian gerbil, Cheung et al 2001 on Cats). First of all it should be specified, but things should be compared with studies on visual (and other sensory stimuli) in anesthetized rats, as there are studies that report latencies on rats' V1.

Added species- description to the discussion. See point 8 for our response to comparisons of specific numbers between studies.

- In line 283-284, the authors state that “neighboring neurons may respond differently to the same level of anesthesia.” This affirmation should be properly discussed and framed in the wide range of literature on the topic.

This topic is now discussed in line 736-741.

- Same thing for the statement at lines 369: “the LFP signature is not only shifted in time, but appears to last longer during anesthesia ”; and at line 430: “activity more correlated when there is no stimulus”. Those are all arguments extensively studied and should be discussed a bit more extensively.

We have added a segment in the discussion about the findings of stronger pair-wise correlation with spontaneous activity and a comparison of the LFP signature. See line 733-735 and 813-821.

- A suggestion for the authors of a paper on neural correlation and synchrony across areas under different levels of anesthesia in rats: Bettinardi et al, Neuroimage 2015.

Reference added to introduction and discussion.

32. General comment for the Discussion: make again reference to specific figure panels while discussing specific results.

We have added references to figures where deemed appropriate in the discussion.

33. Lines 713-714. “on separate pools of an unknown number of units.” What does it man “unknown?” in this paper, is the number of units really not reported? Please double check

With multi-unit activity (MUA) the number of neurons contributing to the signal is unknown. However, since the Durand paper contributes with single-units between separate populations, we have adjusted the sentence to fit with the description of the two studies.

34. Minor problems in the figures:

1A - color legend missing. Categories are indicated on x-axis, thus color legend is not needed.

2E - axis labels missing Corrected.

3A - colorbar label missing Corrected.

3D, left panel - time and amplitude units missing. Corrected.

4B - colorbar label missing. Corrected.

4C - x axis label and color-code label missing. Corrected.

4E - x axis label missing. Corrected.

Reviewer 2:

1. Why did the experimenters chose the three types of anesthesia? It appears for a systematic approach relating to a host of previously generated data under anesthesia, at least the fentanyl condition and also urethane should be added.

We felt that the comparison of interest in the present investigation was the one between the awake and anesthetized state, and that a major limitation to previous investigations on the effect of anesthesia has been the use of a single anesthetic. Instead of comprehensively mapping anesthetic-specific responses, we wanted to provide examples of variations between some commonly used anesthetics that function along different mechanism of action. The rationale for our choice of anesthetics are briefly mentioned in line 125-131. Several investigations have compared responses between many types of anesthetics in the anesthetized state alone, and it is clear from the literature that different types of anesthesia produce different effects on cortical responses. Although adding more types of anesthesia would provide more information about the variability between anesthetics, this was beyond the scope of the current work.

2. The stimulus control is worrisome. The authors compare conditions in which an anesthetized animal was simply placed in front of a screen with situations, in which a mobile awake animal was roaming in front of the screens. They chose to pick the intervals in which the animal was sessile. However, only head position was assessed for this purpose. What about other behaviorally important parameters. What were the animals doing while being sessile? Sleeping, drowsing, grooming, etc.?

We agree with the reviewer that this should have been specified more clearly in the method section. During the experimental sessions the animals were monitored closely and the session was discarded if the rat performed any grooming behavior or displayed signs of drowsing/sleeping. The experiments were performed during the middle of the wake-cycle of the rats, and they were in a dark room aside from the experimental visual stimulation, partly to avoid triggering drowsiness. Hence, we rarely saw drowsing or sleeping rats for our short experimental sessions (120sec). The small size of the box and the short sessions are likely reasons why the rats lie down without sleeping. After several rounds of habituation we often saw this pattern of sessile, quiet, observing behavior (this type of behavior is more rarely seen in larger boxes). We have added a description to our method section of the behavioral monitoring.

In none of the reported situations eye / gaze position was monitored. Nevertheless, the authors present firing rates at a high temporal resolution (milliseconds). What is the effect of different spatial frequencies of the stimulus and receptive field position on the grating on latency and pattern estimations like the one presented in Figs. 2 and 4?

Although eye-tracking would have been optimal and would have opened for a range of more detailed analysis, we do not regard it as essential for the data presented. We would like to highlight that figure 5 A show how the position of the rat in the box would impact the perceived spatial frequency. We calculated that with a maximum movement from one end of the box to the other, the change in the perceived spatial frequency would be limited to one spatial frequency group, e.g. 0.08 c/d to 0.04 c/d. However, since the anesthesia-setup was positioned to a distance that would reflect the middle of the box, the change in spatial frequency would maximally be half of this.

In figure 5 we quantify how latencies in the states vary with differing spatial frequencies. Although there is an overall trend towards lower latencies with increasing spatial frequencies, this is only minimally changed between one spatial frequency group in the awake state. In addition, the latency-change between awake and anesthesia (Fig 5 G) and is all significantly different from anesthesia. Furthermore, to verify our findings, we have compared the spatial frequency response in awake, to the adjacent spatial frequency group in anesthesia (e.g. 0.08 c/d awake compared with 0.04 c/d anesthesia). All comparisons are significantly different.

See answer to point 5 for this issue regarding temporal sequences.

Why wasn't at least the distance varied in anesthetized experiments.

Since the perceived spatial frequency varies so little between the wakeful recordings and anesthesia, and since we map the response characteristics of the various spatial frequencies for each state, we did not find it necessary to vary the distance to the screen for the anesthetized animal. See previous answer and to point 5 for further elaboration.

3. Figure 1: What is meant with anesthesia? An average of all three types? I seriously doubt that this makes sense as in the rest of the paper several significant differences between these regimes are shown. I strongly recommend to abolish all averages across anesthesia regimes from the manuscript!

Despite the different effects of different anesthetics, these differences are still smaller than the difference between awake and anesthesia in general. Thus, we found it informative to have the broad comparison of awake and all types of anesthesia before going into the specific regimes. We also address this issue in our response to reviewer 1 (point 12).

4. Figure 4 and related text: This whole section needs to be reworked completely. It is barely intelligible. The analysis related to patterning has to be succinctly explained here. What is 'average mean (!) spike latency' ???

We agree that this section should be described more clearly. The section has been rewritten and descriptive figures added.

5. In this section the impact of lack of stimulus control is most critical. The authors need to be very meticulous here and argue well which of their results and interpretations still stand when they consider (perhaps systematic) different stimulus conditions in the awake vs. the anesthetized case (eye / whole body movements, distance / angle to the screen etc.).

We appreciate this suggestion, as it would clarify this finding. Although the spatial frequency is likely to vary to a small extent (See answer to point 2) between the states, we would like to show more clearly that this does not impact the reduced sequence stability between wakefulness and anesthesia. Since we have recordings from many of the larger populations with moving animals (experiencing different distance and angle to the stimuli), we have included the single trial rank correlations from these recordings to our figure 4.

Minor comments

1. What is a 16 electrode tetrode array? 4 tetrodes?

Yes, 4 tetrodes per hemisphere, corrected misleading text in line 90.

2. Figure 1: What is the n e.g. in panel B and G? Trials? Spikes? Why is the n only given for one group in panel B?

n is given for all groups in figure 1B. It is 193 units followed between the three states. For figure 1 G, we previously identify the n. But we have added the n to this description as well.

3. Fig. 1D: Abscissa label?

Corrected.

4. Fig. 1G: Show median and iqr (or similar) as well!

Due to the construction of this figure in graphpad prism, it is pretty difficult to display these values on the graph. However, median and standard error of the mean are reported in the result section. In addition, the 95 percentile confidence intervals are reported in the statistics table.

5. Spike types: Demonstrate example waveforms form all three types (BS, NS, triphasic).

Added.

6. Line 346: The statement warrants references!

Reference to Niell & Stryker added.

7. Figure 3D: The abbrev. BS was broad spiking before. What is the purpose of this panel anyway? In a burst suppression situation, enhanced correlation is highly expected!

We will adjust this abbreviation, although we are aware that burst suppression is likely to yield cross-correlations (line 469, “As expected”), we wanted to show that even in the absence of burst suppression pattern, pair-wise correlations are still strong.

8. Fig. 4 is inaccessible! Referencing other papers is not good enough here. Explain all strategies of analysis here in the text!

The result section is rewritten and descriptive figures included.

9. Fig. 5D: Axes labels?

Corrected.

Additional minor changes that I found:

Lines 355-357: completely unclear. Please revise. Maybe also be OK to completely remove and just proceed to the section on “Visually evoked latencies are delayed under anesthesia”.

We use this segment to highlight the transition from basic firing rate properties to changes in temporal aspects of firing, which is the theme of the manuscript. We prefer to keep this segment to maintain this structure.

Lines 347-353 seem inconsistent with line 289-297 that state that only sessile sessions were analyzed

We have added a change to line 310 to adjust this inconsistency.

Line 280: the firing rates were ... (instead of was)

Corrected.

Line 292-293: text is not clear, and seem to disagree with Neil and Stryker results.

The aim of this analysis was to show that the difference between movement and sessile recordings did not affect the reduction in firing rates with anesthesia. We find an insignificant trend due our low n (51) in this analysis, and due to the use of a one-way ANOVA to assess the three-variable comparison. When comparing firing rate with a t-test or non-parametric alternative the firing rate is indeed different between movement and sessile recordings in line with Niell & Stryker (2010).

Line 329: consider the wording such as “had most stable response dynamics” instead of “has the lowest changes in firing rate”. Corrected.

Line 381 ... stimulus onset was ... Corrected.

Line 459: “compared to awake state.” Corrected.
